# High prevalence of multi-drug-resistant bacteria in faecal samples from UK passerine birds

**DOI:** 10.1038/s41598-025-13012-4

**Published:** 2025-08-01

**Authors:** Jenny C. Dunn, Simon R. Clegg

**Affiliations:** 1 School of Life and Environmental Sciences, University of Lincoln, Joseph Banks Laboratories, Green Lane, Lincoln, LN6 7DL UK; 2 Present Address: School of Life Sciences, Keele University, Newcastle-Under-Lyme, Staffordshire, ST5 5DG UK

**Keywords:** Passerine, Drug-resistant, *Enterococcus*, *Salmonella*, *Campylobacter*, *E. coli*, AMR, Antimicrobial resistance, Microbial ecology

## Abstract

Wild birds are a near ubiquitous sight in gardens, offering pleasure to many people through supplementary feeding, song, or other interactions. However, they are also potential carriers of many bacteria, including *Campylobacter* spp., *Salmonella* spp.*, Enterococcus* spp., and *E. coli*; some of these may be resistant to commonly used drugs. This study collected faecal samples from multiple species of UK passerine birds, isolating bacterial pathogens to assess carriage and drug resistances associated with those bacteria. 75% of birds were carrying at least one bacterial species which was multi drug resistant (MDR; resistant to three or more classes of antimicrobial), with 11.6% of birds carrying *Salmonella* spp., 18.9% carrying *Campylobacter* spp., 78% carrying *Enterococcus* spp., and all carrying *E. coli* strains. Many of these strains were shown to be MDR with 70%, 88%, 32% and 59% respectively. Intercontinental migration was shown to be a risk factor for carriage of many of the pathogens, as was an associated with human habitation. Age was also a risk factor with younger birds twice as likely to carry *Campylobacter* spp. than adults, and house sparrows (*Passer domesticus*) and blackbirds (*Turdus merula*) being particularly high-level carriers compared to other species. The high-level carriage and shedding of MDR *E. coli* and other zoonotic pathogens within the faecal samples of multiple species of passerine birds offers a timely reminder of the risks which these bacteria, and their drug resistance profiles may pose to human and animal health in the UK and worldwide. It also shows a level of high environmental contamination, which birds may continue to contribute towards, until our use of antimicrobials, and level of drug-resistant bacteria is decreased. Developing mechanisms for reducing levels of carriage of MDR bacteria in wild bird populations through, for example, increased hygiene around bird feeding practices, may be key in reducing environmental contamination.

**Supplementary Information:**

The online version contains supplementary material available at 10.1038/s41598-025-13012-4.

## Introduction

Antimicrobial resistant (AMR) pathogens are of increasing concern worldwide within both the veterinary and human medical fields making them a One Health concern^1^. Often caused by our overuse and misuse of antimicrobials, AMR bacteria are found in almost every ecosystem investigated, from animals and humans through to soil and water (reviewed by Velazquez-Mesa et al.^2^). This in turn has an effect of increasing both morbidity and mortality in domesticated animals and humans, as well as increases in costs for treatments^3^. Among the most under-investigated systems for AMR carriage is wildlife.

Wildlife pose a major risk for carriage and transmission of AMR bacteria due to their indiscriminate defaecation, and potential to cover large distances, potentially shedding AMR bacteria across wide areas leading to environmental contamination^4^. As treatment of wildlife with any antimicrobial is uncommon, these species act as a good indicator of the levels of contamination of the environment with AMR, with wild animals and birds encountering AMR bacteria through food or water^5^. Worldwide, wild birds offer a large amount of pleasure to people, with as many as 75% of households encouraging them into their gardens with supplementary feeding stations^6^. It has been suggested that AMR in wild birds and other wildlife is associated with anthropogenic activities and environments^5^. However, wild birds are widely considered as potential disseminators of AMR bacteria, due to their tendency to migrate long distances, and to occupy a range of habitats known to be contaminated^7^. Passerine birds within the UK inhabit all available ecosystems, survive on different diets, and have different migration patterns, so they may have a high risk of introduction of novel pathogens into the UK, and subsequent widescale dissemination.

*Escherichia coli* is one of the most commonly tested bacterial species for AMR carriage, due to its simple and rapid isolation, as well as its prevalence as a major component of the gut microbiota in many animal species^8^. In addition, this bacterial species also has a high tendency to both acquire and lose antimicrobial resistance genes. Birds are well known carriers of several different pathogens, including *Campylobacter* spp. and *Salmonella* spp., both of which are major concern for both human and veterinary medicine, causing a range of symptoms from gastrointestinal disease to abortion depending on the specific bacterial species^9,10^. *Enterococcus* spp. is also a major issue within human medicine, being associated with urinary tract infections, septicaemia, and infected wounds, but is also commonly used as a probiotic^11^. Given the potential severity of these pathogens, the presence of AMR poses an increased risk to animal and human health. Indeed, bacterial pathogens of wild avian origin have been suggested to be the cause of outbreaks of disease in humans^9,10^. Many human *Campylobacter* spp., *Salmonella* spp., and *Enterococcus* isolates associated with disease also show some level of AMR. Therefore, studies on bacterial carriage, prevalence and antimicrobial susceptibility are important to inform optimal treatment regimes for human and animal diseases.

Many studies of AMR bacterial carriage in wild birds focus on waterfowl, as these are major risk factors for the transmission of avian influenza^12^. Previous studies have shown AMR *E. coli* in birds from many different countries, as well as the carriage of *Salmonella* spp., *Campylobacter* spp. and *Enterococcus* spp. However, despite their near ubiquitous presence, little is known about the bacterial pathogen carriage of songbirds (Passeriformes) within the UK, which is a major site for migration for many birds across the world^13^, bringing with it the potential risk of introduction of new or novel pathogens, and or AMR genes. In this study, we screen 259 faecal samples from 23 species of passerine birds to quantify the prevalence of four different bacterial pathogens and assess their susceptibility to a range of different antimicrobials from different classes which are used to treat both animal and human clinical cases. We then test for host and ecological associations with infection by each pathogen to elicit potential drivers or risk factors for infection.

## Methods

### Sites and sample collection

Faecal samples were collected from wild birds caught as part of standard bird ringing activities at three different sites. One site, near Braintree, Essex, UK (51°53′24.8″N, 0°33′18.3″E) was a residential garden of approximately 0.75 ha surrounded by arable farmland, where ten birdfeeders were provided to encourage birds into the garden. Feeders were kept full year-round, with provided food including sunflower hearts (*Helianthus annuus*), peanuts (*Arachis hypogaea*), a bird seed mix (dominated by wheat (*Triticum aestivum*), and nyjer seed (*Guizotia abyssinica*). The second site, near Potterhanworth, Lincolnshire, UK (53°11′02.5″N, 0°25′21.5″W) was a small woodland copse surrounded by arable farmland, with wheat provided year-round to feed gamebirds (mostly ring-necked pheasants (*Phasianus colchicus*)). The third site, near Glentham, Lincolnshire, UK (53°24′03.8″N, 0°29′37.5″W) consisted of three small lakes bordered by scrub (mostly hawthorn (*Crataegus* sp.) and blackthorn (*Prunus spinosa*) surrounded by arable farmland, where no supplementary food was provided.

At each site, birds were captured using mist nets on days that were dry and still. Birds were caught on 14 occasions per site between June–August 2022 and fitted with an individually numbered BTO metal ring before being aged and sexed where possible according to plumage characteristics^14^, measured (maximum wing chord measured using a slotted wing rule, ± 0.5 mm) and weighed using a digital balance (± 0.1 g). Faecal samples were collected following release of the bird, from the inside of the clean and disinfected cotton bird bag within which the bird was kept prior to processing, stored at ambient temperature in the field (up to 6 h) and then stored at 4 °C until processing. This study received ethical approval from the University of Lincoln Animal Ethics Committee, reference LEAS3818. The study is reported in accordance with ARRIVE guidelines where appropriate. All methods were performed in accordance with the relevant guidelines and regulations. No clinical signs of ill health were observed in any of the sampled birds.

### Sample preparation in the laboratory

From each faecal sample, 0.1 g was resuspended in 900 µl of sterile physiological saline (Melford, UK) before being plated onto agar and incubated for 24 h at 37 °C.

### Bacterial isolation for all samples

For each sample the isolation of *E. coli, Salmonella* spp., *Enterococcus* spp. and *Campylobacter* spp. was performed.

Firstly, *E. coli* was isolated using MacConkey agar (Oxoid, UK), and three suspected *E. coli* colonies were selected from each plate and resuspended in PBS before being subcultured onto Columbia agar plates (Oxoid, UK) and incubated for 24 h at 37 °C to obtain a monoculture. Bacterial strains were identified using Gram staining and PCR targeting the 16S rRNA gene to confirm that isolates were *E. coli*^15^. This allows for detection of all *E. coli* strains, and does not differentiate Shiga toxigenic *E. coli* (STEC) and other toxinogenic strains (Sabat et al. 2000). A selection of these were subjected for sequencing to confirm PCR specificity (data not shown). All strains were stored with nutrient broth (Oxoid, UK) and glycerol (Sigma Aldrich UK) at a ratio of 80:20 at − 80 °C until further analysis.

The identification of *Salmonella* spp. was based on ISO 6579-1:2017. Firstly, 0.1g of each faecal sample was pre-enriched in 1:10 buffered peptone water (ThermoFisher Scientific, UK) before incubation at 37 °C for 18–20 h. From this pre-enrichment, 100µl were transferred onto semi solid modified Rappaport Vassiliadis (Difco, UK) before incubation for 48 h at 41.5 °C. Colonies suggestive of *Salmonella* spp. were further inoculated onto Xylose-Lysine Deoxycholate (ThermoFisher Scientific, UK) and chromogenic agar specific for detection of C8-esterase activity (ASAP, bioMerieux, Marcy l’Étoile, France) and incubated at 37 °C for 48 h. Isolates were confirmed to be *Salmonella* spp. by PCR targeting the *invA* gene^16^, and serotyped using the antigenic agglutination method with specific antisera according to the White-Kauffmann-Le Minor scheme (ISO 6579-1:2017).

For the identification of *Enterococcus* spp., the faecal dilutions prepared previously for *E. coli* isolations were inoculated onto Slanetz and Bartley agar (Oxoid, UK) and incubated at 37 °C for 48 h. A single isolate (red, maroon or pink coloured colony) was removed from the plate and subcultured in LB broth (Melford, UK) before incubation at 37 °C for 48 h. DNA from a small aliquot of this culture was extracted using a boil preparation^17^ and was confirmed to be *Enterococcus* spp. using PCR targeting the *tuf* gene^18^. Speciation was carried out using the methods described by Jackson et al.^19^.

Isolation of *Campylobacter* spp. was performed using the ISO 10272-1:2017 as described previously^20^. Briefly, modified charcoal cefoperazone deoxycholate (mCCDA) and Preston agar (Oxoid, UK) were both streaked with the diluted samples, and incubated in a microaerophilic environment at 41.5°C for 48 h. Colonies indicative of *Campylobacter* spp. were examined by PCR for genus, and species confirmation using various genes^21^.

All PCR primers used in the study can be found in supplementary Table S1.

### Antimicrobial susceptibility testing for all isolates

Antimicrobial susceptibility for *E. coli* was performed using the Kirby-Bauer disk diffusion method according to the European Committee on Antimicrobial Susceptibility Testing (EUCAST) guidelines^22^. Isolates were recovered from the frozen stocks and spread onto Columbia Agar with 5% sheep blood (Scientific Laboratory Supplies, UK) before being suspended in 0.8% saline solution to obtain a turbidity of 0.5 McFarland units^23^. This inoculum was transferred onto Mueller- Hinton Agar (Oxoid, UK) and antimicrobial discs placed on the surface. In total, 22 antimicrobials from 11 different classes were tested based on previous studies^24,25^ to allow for comparison. Plates were incubated for 18–20 h at 36 °C before susceptibility or resistance was assessed through growth inhibition diameter according to EUCAST breakpoints based on 2024 guidelines (EUCAST). Exceptions to this were ceftiofur, enrofloxacin and tetracycline which were evaluated based on previous studies^26,27^. Multidrug resistance (MDR) was determined if an isolate was shown as fully resistant to at least one antimicrobial agent in three or more antimicrobial classes^27,28^. *E. coli* ATCC 11755 was used as a reference strain.

Antimicrobial resistance testing for *Salmonella* spp. was determined using ISO 20776-1:2006 using broth microdilution testing performed using Mueller Hinton Broth (Oxoid, UK) to allow for determination of the minimum inhibitory concentration (MIC). All antimicrobials were tested at concentrations detailed in Table 1, and results were interpreted using EUCAST breakpoints 2024^29,30^. For this, 96 well plates (Sarstedt, UK) were inoculated, bacterial broth culture diluted to the correct optical density and antimicrobials serially diluted to the required concentrations before being incubated at 37 °C in a plastic bag with a paper towel moistened with sterile water. Each plate included a negative control (no bacteria) and a positive control row (no antimicrobial). Using these data, they were assigned a resistant, intermediate or susceptible phenotype. Isolates were tested against ten different antimicrobials from seven different classes (Table 1).

**Table 1:**
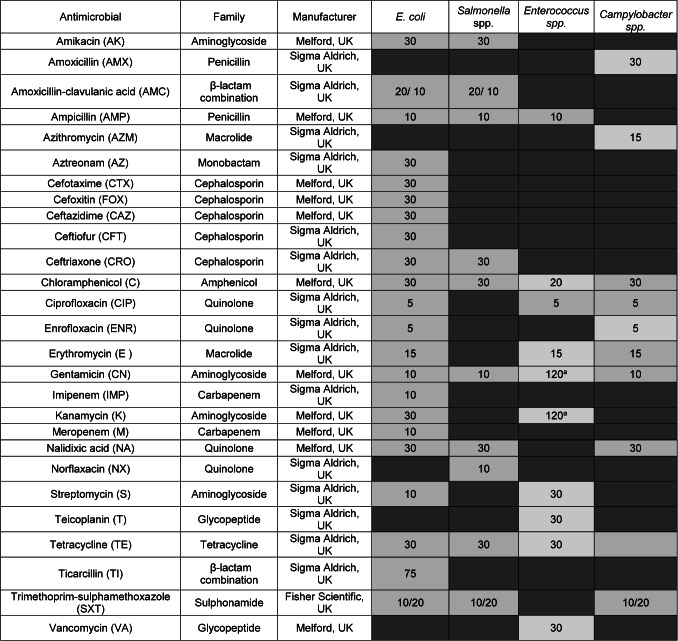
Antimicrobials used in the study along with family and manufacturer of the antimicrobials, and abbreviations used subsequently in brackets. Cells in light grey are the antimicrobials used in the study, along with the concentration used (in µg/ml), those in dark grey were not used to test for that bacterial species. Resistance was determined based on EUCAST or CLSI guidelines 2024 and the clinical breakpoint is the concentration shown here. For *Salmonella* spp., *Enterococcus* spp., and *Campylobacter* spp., all antimicrobials were tested at the concentration range of 250ug/ml to 0.5 ug/ml, with the exception of those marked in the table with ^a^, where a different higher range was used due to the higher clinical breakpoint (760 ug/ml to 2ug/ml). *E. coli* was tested using antimicrobial impregnated discs at the concentrations stated.

Further MIC analysis testing was undertaken for *Enterococcus* spp., using a similar methodology as that used for *Salmonella* spp., following the CLSI guidelines (antimicrobials are shown in Table 1) and were in line with previous studies^31^ to allow for comparisons. Using this data, they were assigned a resistant, intermediate or susceptible phenotype. For controls, the reference strains used were *E. faecalis* ATCC 29212 and *Staphylococcus. aureus* ATCC 29213. Analysis compared the breakpoints to those with the CLSI standards or the national antimicrobial resistance monitoring system.

Broth microdilution was also used for all *Campylobacter* isolates (Luber et al., 2003). Using sensitive *Campylobacter* EUCAMP2® plates (ThermoFisher Scientific, UK) according to manufacturers instructions, each isolate was tested against 10 different antimicrobials (Table 1) and assigned a resistant, intermediate or susceptible phenotype based on the epidemiological cut off values established by EUCAST 2024.

### Statistical analyses

All statistical analyses were conducted in R version 4.3.1 “Beagle Scouts” for Mac^32^. Two binomial general linear models were constructed to test for associations between each of environmental variables and host ecological variables on the presence and MDR status of each bacterium (fourteen models in total; all birds were carrying *E. coli* so no models were constructed to test for associations with *E. coli* presence). For each model, the binomial response variable was the presence or absence of either the bacterium, or MDR. For the environmental model, fixed factors comprised Site (a 3-level factor), Species (a 16-level factor), Age (a two-level factor of juvenile (hatched during the calendar year of capture) or adult (hatched prior to this) and day (a continuous variable). For the ecological models, fixed factors comprised Migrant status (resident or long-distance migrant; species that may undertake short-distance migration were classified as resident for the purposes of this analysis), whether the species was associated with human habitation (a two-level factor of Yes or No), whether the species was granivorous (Yes or No) or insectivorous (Yes or No) and the number of food types used by the species (a continuous variable). Long-distance migrants were blackcap *Sylvia atricapilla*, chiffchaff *Phylloscopus collybita*, garden warbler *Sylvia borin*, lesser whitethroat *Sylvia curruca*, reed warbler *Acrocephalus scirpaceus*, sedge warbler *Acrocephalus schoenobaenus*, willow warbler *Phylloscopus trochilus* and whitethroat *Sylvia communis*. Host ecological data were extracted from^33^ at the species level. Models were simplified by removing the least significant term in turn (as determined by likelihood ratio tests) until either all remaining terms in the model were significant at p < 0.1, or only the null model remained. Terms were interpreted as being significantly associated with the response variable when p < 0.05.

## Results

259 faecal samples from 23 bird species were screened for *Salmonella* spp., *Campylobacter* spp., *Enterococcus* spp. and *E. coli*, along with antimicrobial resistance profiles. Species sampled were Eurasian blackbird *Turdus merula* (n = 6), blackcap *Sylvia atricapilla* (n = 25), blue tit *Cyanistes caeruleus* (n = 29), bullfinch *Pyrrhula pyrrhula* (n = 3), chaffinch *Fringilla coelebs* (n = 30), chiffchaff *Phylloscopus collybita* (n = 3), dunnock *Prunella modularis* (n = 14), garden warbler *Sylvia borin* (n = 1), European goldfinch *Carduelis carduelis* (n = 4), great tit *Parus major* (n = 13), greenfinch *Carduelis chloris* (n = 4), house sparrow *Passer domesticus* (n = 40), lesser whitethroat *Sylvia curruca* (n = 1), long-tailed tit *Aegithalos caudatus* (n = 3), Common reed bunting *Emberiza schoeniclus* (n = 5), Eurasian reed warbler *Acrocephalus scirpaceus* (n = 10), European robin *Erithacus rubecula* (n = 24), sedge warbler *Acrocephalus schoenobaenus* (n = 3), song thrush *Turdus philomelos* (n = 1), common whitethroat *Sylvia communis* (n = 15), willow warbler *Phylloscopus trochilus* (n = 5), wren *Troglodytes troglodytes* (n = 16) and yellowhammer *Emberiza citrinella* (n = 4).

### *Salmonella*

*Salmonella* spp. was identified from 30 (11.6%) of 259 faecal samples using PCR. Twelve serovars of *Salmonella* were isolated (Table 2), the most common of which, *Salmonella enterica* serovar Typhimurium, was isolated from eleven individuals from seven bird species (Table 2). MDR was identified in 21 (70%) of the 30 positive samples (Table 2). MDR was identified in the serovars Agona (100%, n = 2), Havana (100%, n = 1), Meleagridis (100%, n = 2), Muenchen (50%, n = 1), Muenster (100%, n = 2), Newport (50%, n = 2), Panama (50%, n = 2), Parathyphi B var. Java (33%, n = 3), Rissen (100%, n = 1) and Typhimurium (82%, n = 11; Table 2).

**Table 2:** The number of individuals from each host species from which each *Salmonella* strain was isolated. The number of individuals with strains showing MDR is provided in parentheses.

	Host species										
*Salmonella* strain	Blackbird	Blackcap	Blue tit	Chaffinch	Chiffchaff	Dunnock	Goldfinch	House sparrow	Robin	Whitethroat	Wren
Agona								2 (2)			
Havana											1 (1)
Heidelberg	1 (0)										
Meleagris						1 (1)		1 (1)			
Montevideo									1 (0)		
Muenchen		1 (1)	1 (0)								
Muenster		1 (1)	1 (1)								
Newport	1 (0)	1 (1)									
Panama								2 (1)			
Paratyphi B var. Java						1 (0)	1 (1)		1 (0)		
Rissen							1 (1)				
Typhimurium		2 (2)	1 (1)	2 (1)	1 (1)			3 (2)		1 (1)	1 (1)

**Fig. 1:**
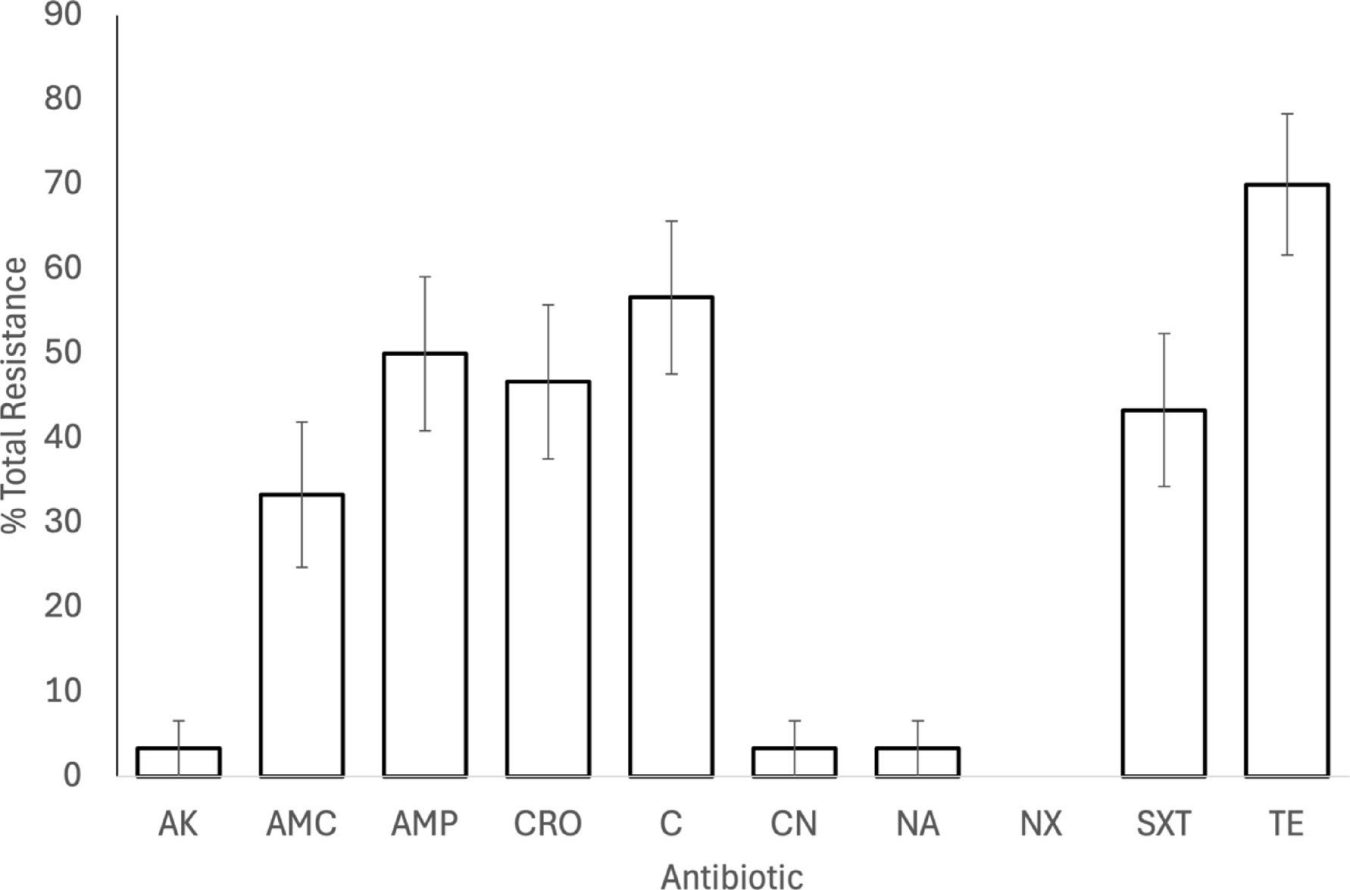
Percentage total antimicrobial resistance in isolated *Salmonella* samples (n = 30). Bars show mean ± 1 SE, full antimicrobial names can be found in Table 1. Full resistance data are provided in Supplementary Table S2. Key: AK: Amikacin, AMC: Amoxycillin-clavulanic acid, AMP: Ampicillin, CRO: Ceftriaxone, C: Chloramphenicol, CN: Gentamycin, NA: Nalidixic acid, NX: Norfloxacin, SXT: Trimethoprim-sulphamethoxazole, TE: Tetracycline.

Resistance was highest to tetracycline (n = 21, 70%), chloramphenicol (n = 17, 57%) and ampicillin (n = 15, 50%), with 50% or more of samples showing complete resistance (Fig. 1; Table S2). Resistance was lowest to nalidixic acid, with no samples showing complete resistance and three samples (10%) showing intermediate levels of resistance, followed by amikacin, where one sample (3%) showed complete resistance and no samples showed intermediate resistance (Fig. 1; Table S2).

Neither the presence of *Salmonella*, nor the presence of MDR *Salmonella* within positive samples, differed between sites, or between adult and juvenile birds, and did not vary with Julian day (Table 3). The presence of *Salmonella* did not differ between species (Table 3a; this analysis was not conducted for MDR *Salmonella* due to small sample sizes), and none of diet, migration strategy or breeding presence within human settlements influenced the presence of *Salmonella* (Table 4). However, all intercontinental migrants with *Salmonella* were carrying strains with MDR (Migrants: 100% MDR [n = 7], non-migrants: 59% MDR [n = 22]; Table 4), and birds carrying non-MDR *Salmonella* had higher diet diversity than those carrying MDR *Salmonella* (non-MDR *Salmonella*: 2.22 ± 0.15 food types; MDR *Salmonella*: 1.75 ± 0.10 food types; Table 4).

**Table 3:** Results of binomial generalised linear models testing the effect of environmental variables on the presence of a) *Salmonella* spp., *Campylobacter* spp. and *Enterococcus* spp. and b) MDR *Salmonella* spp., MDR *Campylobacter* spp., MDR *Enterococcus* spp. and MDR *E. coli* in avian faecal samples. Terms interpreted as significantly influencing the response variable are highlighted in bold; marginally significant terms retained in the final model are italicised. Dev = deviance, df = degrees of freedom, p = *p* value. “- “ indicates that this variable was not tested due to small sample sizes within categories.

a	*Salmonella s*pp.			*Campylobacter* spp.			*Enterococcus* spp.					
	Dev	df	*p*	Dev	df	*p*	Dev	df	*p*			
Species	21.700	15, 239	0.116	**35.294**	**15, 238**	**0.002**	17.272	15, 238	0.303			
Age	0.006	1, 222	0.938	**4.616**	**1, 223**	**0.032**	1.284	1, 239	0.257			
Julian day	1.500	1, 223	0.221	0.177	1, 222	0.177	1.045	1, 223	0.307			
Site	0.143	2, 220	0.931	0.781	2, 220	0.677	0.394	2, 222	0.821			

b	MDR *Salmonella s*pp.			MDR *Campylobacter* spp.			MDR *Enterococcus* spp.			MDR *E. coli*		
	Dev	df	*p*	Dev	df	*p*	Dev	df	*p*	Dev	df	*p*
Species	-	-	-	8.514	14,45	0.861	23.978	21, 197	0.294	19.360	15, 239	0.198
Age	0.731	1, 28	0.393	-	-	-	1.422	1, 198	0.233	0.543	1, 221	0.461
Julian day	0.161	1, 27	0.688	0.064	1, 46	0.801	**7.312**	**1, 199**	**0.007**	1.438	1, 222	0.231
Site	0.145	2, 26	0.930	*4.743*	*2, 48*	0.093	1.441	2, 176	0.487	0.671	1, 224	0.413

**Table 4:** Results of binomial generalised linear models testing the effect of host species ecological traits on the presence of a) *Salmonella* spp., *Campylobacter* spp. and *Enterococcus* spp. and b) MDR *Salmonella* spp., MDR *Campylobacter* spp., MDR *Enterococcus* spp. and MDR *E. coli* in avian faecal samples. Terms interpreted as significantly influencing the response variable are highlighted in bold; marginally significant terms retained in the final model are italicised. Dev = deviance, df = degrees of freedom, *p* = *p* value.

a	*Salmonella s*pp.			*Campylobacter* spp.			*Enterococcus* spp.					
	Dev	df	*p*	Dev	df	*p*	Dev	df	*p*			
Human habitation	0.496	1, 254	0.481	**13.100**	**1, 253**	** < 0.001**	1.976	1, 253	0.160			
Granivorous	1.162	1, 253	0.281	*3.250*	*1, 253*	0.072	1.624	1, 254	0.203			
Insectivorous	0.561	1, 252	0.454	0.129	1, 251	0.720	0.939	1, 252	0.332			
Diet diversity	0.592	1, 251	0.442	0.051	1, 252	0.821	0.025	1, 250	0.873			
Migratory (Y/N)	0.104	1, 250	0.747	0.015	1, 250	0.903	0.589	1, 251	0.443			

b	MDR *Salmonella s*pp.			MDR *Campylobacter* spp.			MDR *Enterococcus* spp.			MDR *E. coli*		
	Dev	df	*p*	Dev	df	*p*	Dev	df	*p*	Dev	df	*p*
Human habitation	0.200	1, 25	0.655	0.691	1, 48	0.406	1.861	1, 198	0.173	0.571	1, 251	0.450
Granivorous	*3.68*	*1, 26*	0.055	1.361	1, 46	0.243	0.213	1, 196	0.645	0.916	1, 252	0.339
Insectivorous	0.001	1, 24	0.999	0.858	1, 45	0.384	*2.836*	*1, 199*	0.092	1.408	1, 253	0.235
Diet diversity	**11.353**	**1, 26**	** < 0.001**	0.872	1, 44	0.350	0.388	1, 197	0.533	**6.354**	**1, 254**	**0.012**
Migratory (Y/N)	**9.00**	**1, 26**	**0.003**	1.067	1, 47	0.301	0.021	1, 195	0.884	0.037	1, 250	0.848

### *Campylobacter* spp.

*Campylobacter* spp. were identified from 49 (18.9%) of 259 faecal samples using PCR (Table 5). Species-specific PCRs identified 3 *C. coli* infections (6.1%); 22 *C. lari* infections (44.9%) and 24 *C. jejuni* infections (49.0%); no birds were infected by multiple *Campylobacter* species. Antimicrobial resistance to at least three classes of antimicrobial was identified in 43 (88%) of the 49 positive samples (3 (100%), *C. coli* infections; 20 (91%), *C. lari* infections; 20 (83%) and *C. jejuni* infections; Table 5).

**Table 5:** The number of individuals from each host species from which *Campylobacter* spp. was isolated. The number of individuals with strains showing MDR is provided in parentheses. Bullfinch (n = 3), dunnock (n = 14), garden warbler (n = 1), lesser whitethroat (n = 1), long-tailed tit (n = 3), reed bunting (n = 5), sedge warbler (n = 3) and yellowhammer (n = 4) were tested but found to be negative for *Campylobacter*; these species are not included.

Host species	*C. coli*	*C. lari*	*C. jejuni*
Blackbird (n = 6)		1 (1)	2 (2)
Blackcap (n = 25)		2 (2)	1 (0)
Blue tit (n = 29)		4 (4)	1 (1)
Chaffinch (n = 30)	1 (1)	2 (2)	
Chiffchaff (n = 3)			1 (1)
Goldfinch (n = 4)		1 (1)	
Great tit (n = 13)			1 (1)
Greenfinch (n = 4)			1 (1)
House sparrow (n = 40)	1 (1)	7 (5)	11 (8)
Reed warbler (n = 10)			1 (1)
Robin (n = 24)	1 (1)		3 (3)
Song thrush (n = 1)		1 (1)	
Whitethroat (n = 15)		2 (2)	1 (1)
Willow warbler (n = 5)			1 (1)
Wren (n = 16)		2 (2)	

**Fig. 2:**
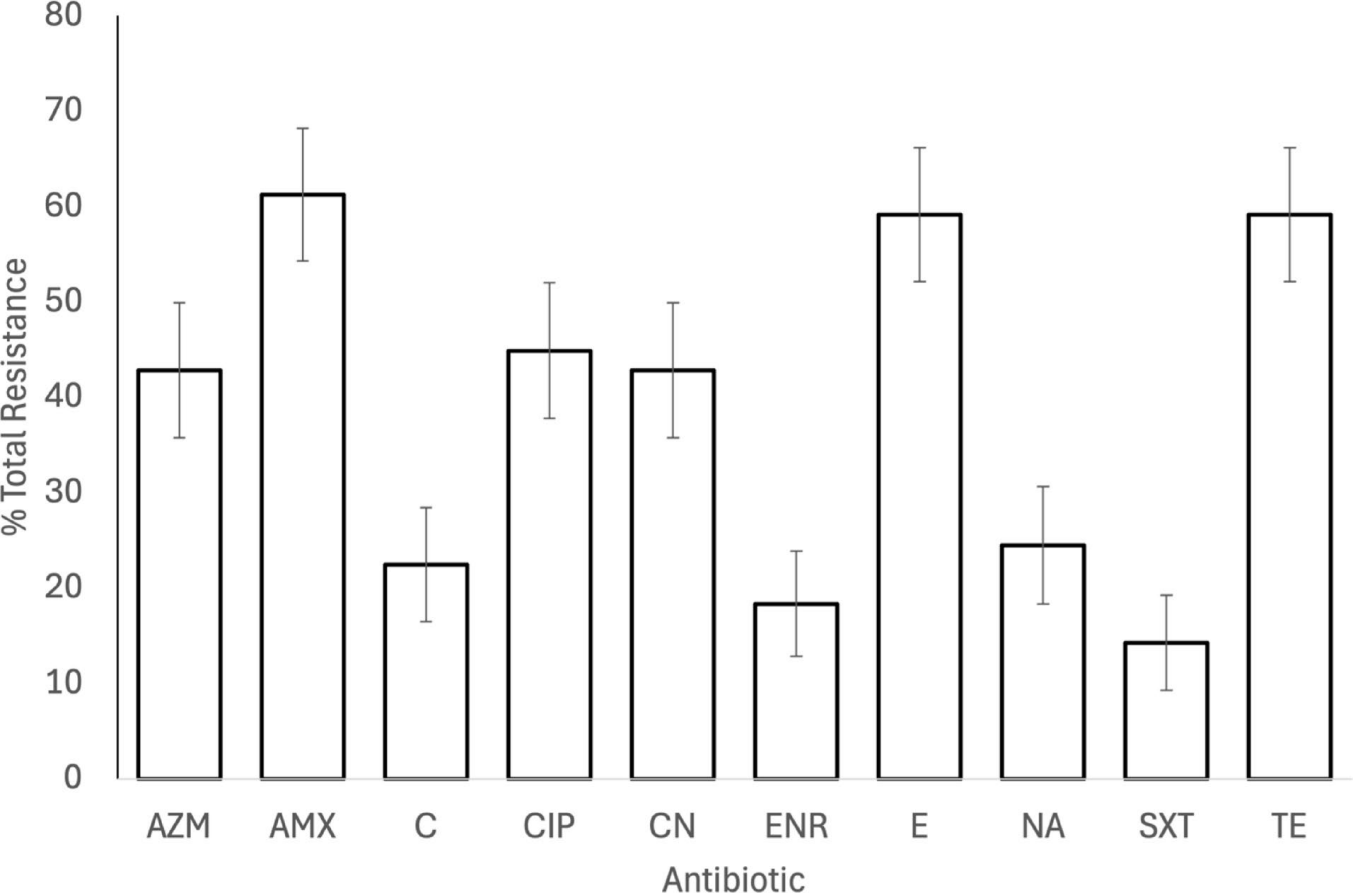
Percentage total antimicrobial resistance in isolated *Campylobacter* samples (n = 49). Bars show mean ± 1 SE, full antimicrobial names can be found in Table 1. Full resistance data are provided in Supplementary Table S3. Key: AZM- Axithromycin, AMX: Amoxycillin, C: Chloramphenicol, CIP: Ciprofloxacin, CN: Gentamycin, ENR: Enrofloxacin, E: Erythromycin, NA: Nalidixic acid, SXT: Trimethoprim-sulphamethoxazole, TE: Tetracycline.

Resistance was highest to amoxicillin (n = 30, 61%), tetracycline (n = 29, 59%) and erythromycin (n = 29, 59%), with over 50% of samples showing full or partial resistance to all tested antimicrobials (Table S3). Resistance was lowest to trimethoprim-sulfamethoxazole, with 7 samples (14%) showing complete resistance, and a further eight samples (16%) showing intermediate resistance, and to enrofloxacin, where nine samples (18%) showed complete and 8 samples (16%) showed intermediate resistance (Fig. 2; Table S3).

**Fig. 3:**
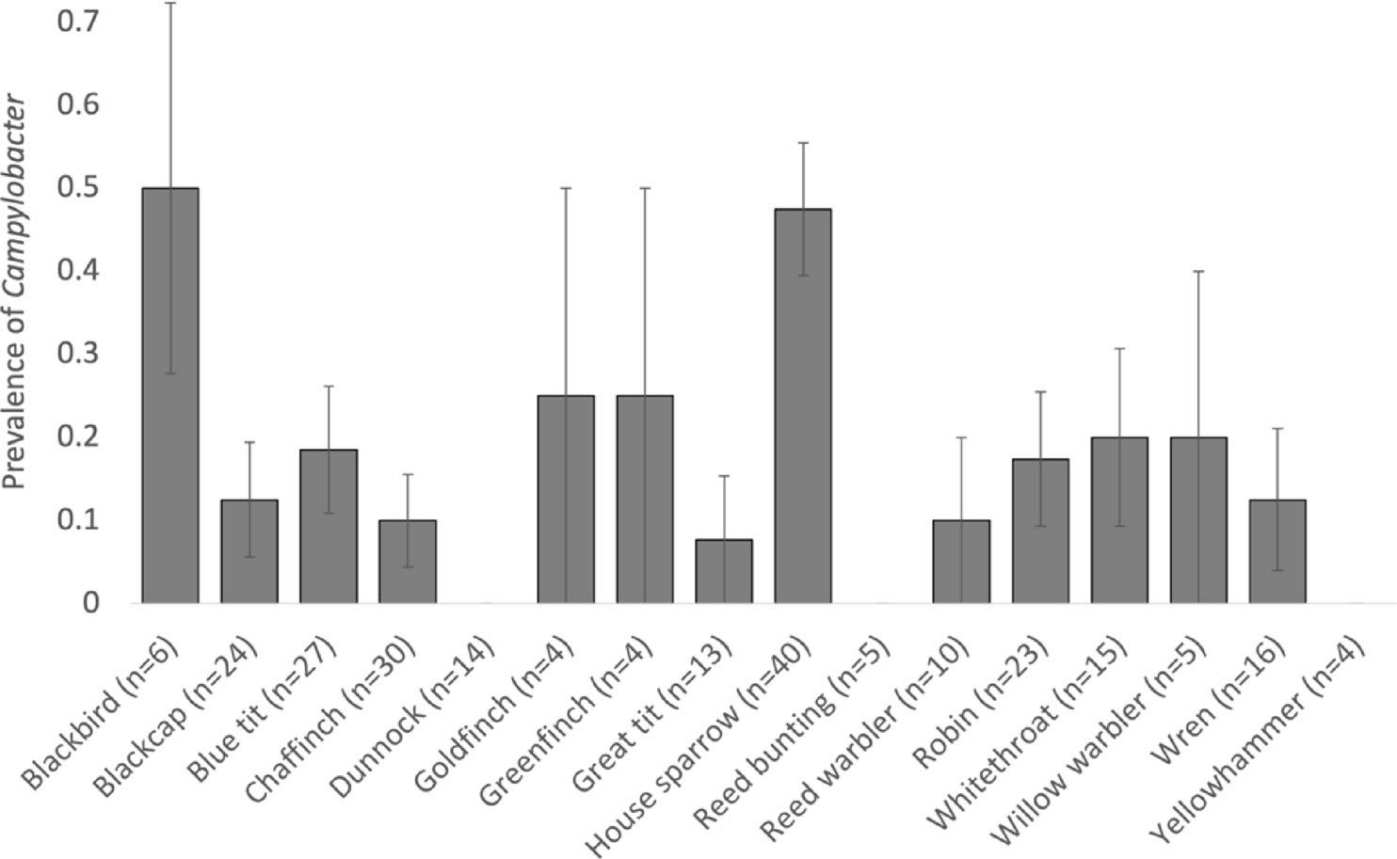
Differences between species in *Campylobacter* prevalence. Bars show mean ± 1 SE.

The prevalence of *Campylobacter* differed between species (Table 3a; Fig. 3), and juvenile birds were more than twice as likely to be infected as adults (juveniles: 21.5 ± 2.9% prevalence; 8.6 ± 4.8% prevalence). The presence of MDR *Campylobacter* spp. differed marginally between sites, with 100% (n = 9) of positive samples from the fed farmland site being resistant to at least three classes of antimicrobial (Table 3). The lowest prevalence of MDR was at the Essex garden site (78% of positive samples; n = 23), with 94% (n = 17) of positive samples at the unfed farmland site showing MDR.

The prevalence of *Campylobacter* was higher in birds associated with human habitation (Table 4; associated with human habitation: 69 ± 7% [n = 49]; not associated with human habitation: 44 ± 3% [n = 206]). The prevalence of MDR *Campylobacter* was not associated with any host ecological traits (Table 4).

### *Enterococcus* spp.

*Enterococcus* spp. was identified from 203 (78%) of 259 faecal samples using PCR (Table 6). Species-specific PCRs identified 7 (3.4% of positives) *E. casseliflavus* infections, 10 (4.9%) *E. durans* infections, 86 (42.4%) *E. faecalis* infections, 88 (43.3%) *E. faecium* infections and 12 (5.9%) *E. hirae* infections. MDR was identified in 65 (32%) of positive infections. No MDR was found in *E. casseliflavus*, *E. durans* or *E. hirae*, but 44 (51.2%) of *E. faecalis* infections and 21 (23.9%) *E. faecium* infections showed MDR (Table 6).

**Table 6:** The number of individuals from each host species from which *Enterococcus* spp. was isolated. The number of individuals with strains showing MDR is provided in parentheses.

	*E. casseliflavus*	*E. durans*	*E. faecalis*	*E. faecium*	*E. hirae*
Blackbird			3 (2)		
Blackcap	1 (0)	2 (0)	10 (5)	6 (1)	
Blue tit	1 (0)	1 (0)	13 (7)	7 (2)	2 (0)
Bullfinch		1 (0)		2 (0)	
Chaffinch	1 (0)		10 (6)	11 (1)	2 (0)
Chiffchaff			1 (0)		
Dunnock		1 (0)	5 (4)	6 (2)	
Goldfinch			1 (1)	1 (0)	1 (0)
Great tit		1 (0)	3 (2)	3 (0)	1 (0)
Greenfinch			1 (0)	3 (0)	
House sparrow	2 (0)	3 (0)	10 (6)	13 (9)	3 (0)
Lesser whitethroat				1 (0)	
Long-tailed tit			1 (1)	1 (0)	
Reed bunting			3 (1)	2 (0)	
Reed warbler	1 (0)		2 (2)	3 (0)	
Robin			11 (3)	7 (2)	1 (0)
Sedge warbler			1 (1)	1 (0)	
Song thrush				1 (0)	
Whitethroat			5 (3)	7 (2)	1 (0)
Willow warbler				3 (0)	
Wren	1 (0)	1 (0)	4 (1)	7 (2)	1 (0)
Yellowhammer			1 (0)	3 (0)	

**Fig. 4:**
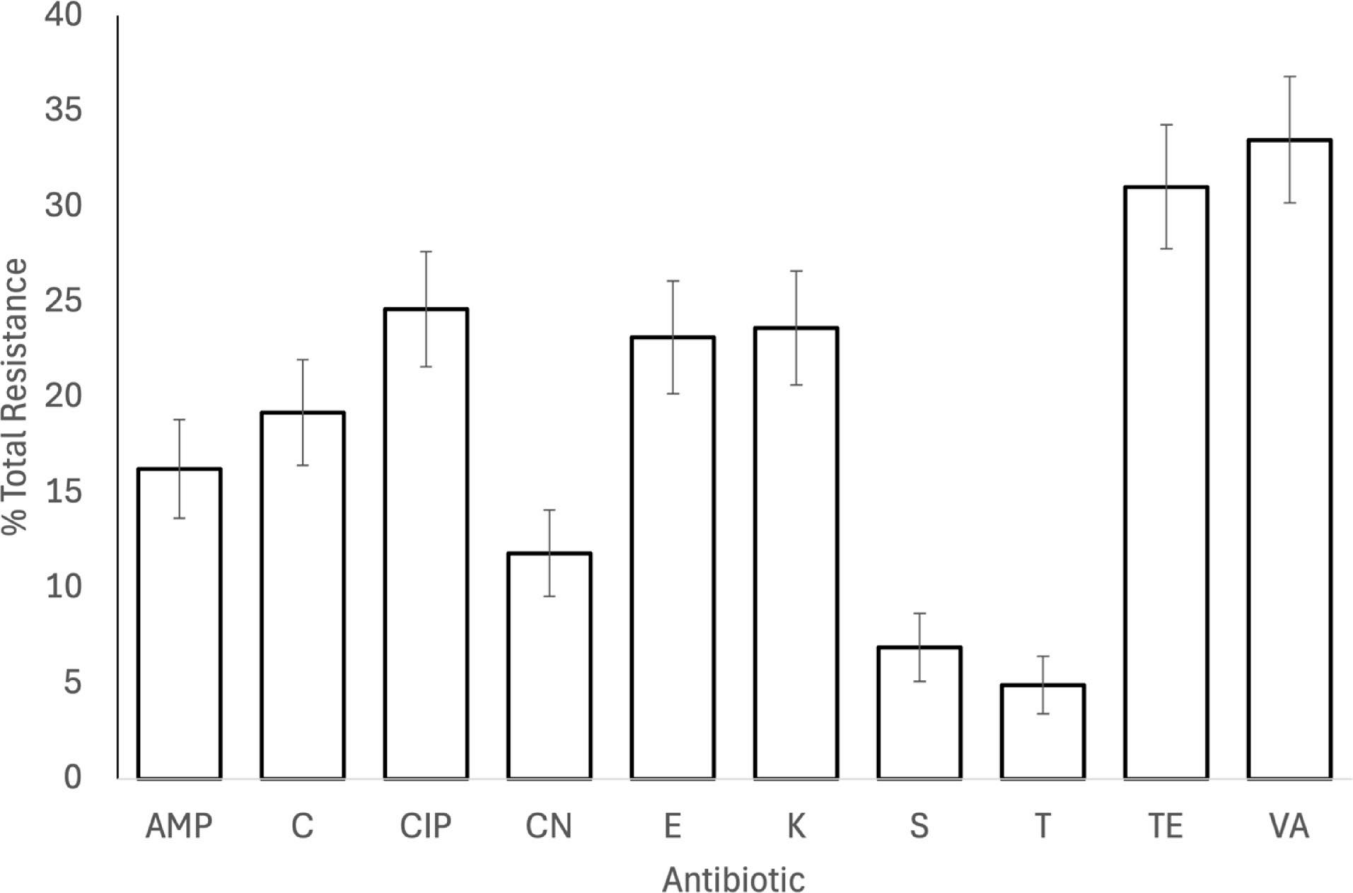
Percentage total antimicrobial resistance in isolated *Enterococcus* samples (n = 203). Bars show mean ± 1 SE, full antimicrobial names can be found in Table 1. Full resistance data are provided in Supplementary Table S4. Key: AMP: Ampicillin, C: Chloramphenicol CIP: Ciprofloxacin, CN: Gentamycin, E: Erythromycin, K: Kanamycin, S: Streptomycin, T: Teicoplanin, TE: Tetracycline, VA: Vancomycin.

Resistance was highest to vancomycin (n = 68, 33%) and tetracycline (n = 63, 31%), and lowest to teicoplanin (n = 10, 5%) and streptomycin (n = 14, 7%) (Fig. 4; Table S4).

None of Julian day, age, species or site were associated with the presence of *Enterococcus* (Table 3). However, the prevalence of MDR *Enterococcus* declined throughout the season (Table 3). None of diet diversity, migratory status, human habitation or granivorous status were associated with the prevalence of either *Enterococcus* or MDR *Enterococcus* in infected birds, but birds that ate invertebrates had a marginally higher prevalence of MDR *Enterococcus* than birds that did not eat invertebrates (invertebrates: 33.2 ± 3% [n = 190]; no invertebrates: 10.0 ± 10.0% [n = 10]; Table 4).

### *E. coli*

**Fig. 5:**
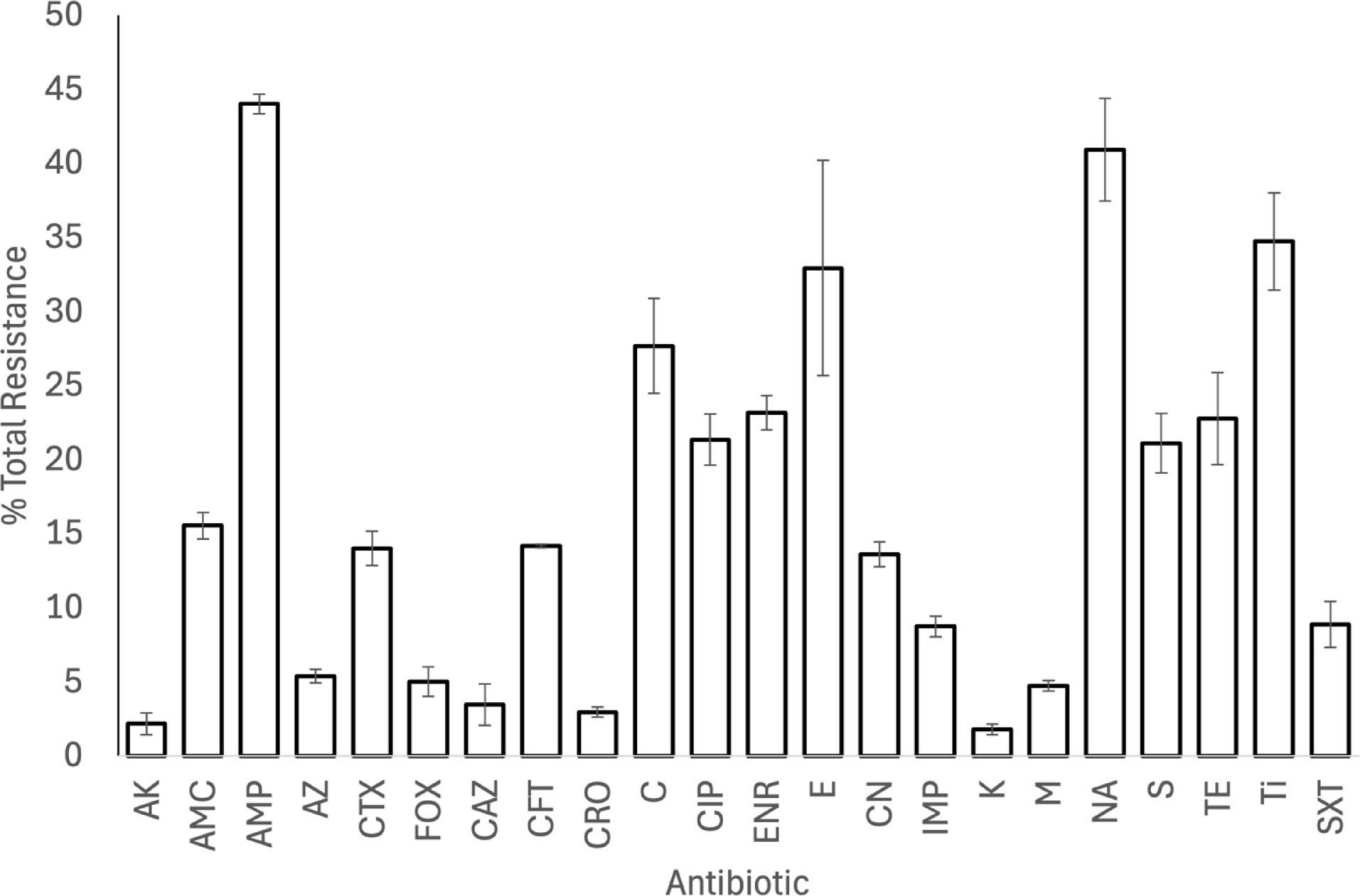
Percentage total antimicrobial resistance in isolated *E. coli* samples (n = 259). Bars show mean ± 1 SE, full antimicrobial names can be found in Table 1. Full resistance data are provided in Supplementary Table S5. Key: AK: Amikacin, AMC: Amoxycillin-clavulanic acid, AMP: Ampicillin, AZ: Aztreonam, CTX: Cefotaxime, FOX: Cefoxitin, CAZ: Ceftazidime, CFT: Ceftiofur, CRO: Ceftriaxone, C: Chloramphenicol, CIP: Ciprofloxacin, ENR: Enrofloxacin, E: Erythromycin, CN: Gentamycin, IMP: imipenem, K: Kanamycin, M: Meropenem, NA: Nalidixic acid, S: Streptomycin, TE: Tetracycline, Ti: Ticarcillin, SXT: Trimethoprim-sulphamethoxazole.

All 259 avian faecal samples were positive for *E. coli*; 153 (59%) of these were resistant to at least three classes of antimicrobial (Fig. 5; Table S5). Resistance was highest to ampicillin (n = 114, 44%) and nalidixic acid (n = 106, 41%) and lowest to kanamycin (n = 5, 2%) and amikacin (n = 6, 2%; Fig. 5; Table S5). The presence of MDR *E. coli* did not differ between sites, species, or age classes of bird, or with Julian day (Table 3). Birds with MDR *E. coli* had a higher diet diversity that those without (with MDR *E. coli*: 1.98 ± 0.04 food types [n = 150]; without MDR *E. coli*: 1.81 ± 0.05 food types [n = 105]); no other host ecological traits were associated with infection by MDR *E. coli* (Table 4).

## Discussion

This study is one of very few that investigates multiple pathogens from within the same animal, with many other studies reporting results separately making comparisons difficult. The high levels of antimicrobial resistant bacteria for all species tested (*Campylobacter, Salmonella, Enterococcus* and *E. coli*) is of concern, but perhaps not a surprise given the huge rise in use of AMR seen globally in many different species. Although wildlife AMR is tested much less frequently than companion or livestock AMR, these animals—especially birds due to the long distances which they travel—can provide a good measure of environmental AMR contamination with both AMR bacteria, and resistance genes^34^. This study shows that many bacteria in wild passerine birds acquire resistance from an unknown source, possibly food, or water, and cannot be easily treated if they were to cause disease in birds or other animals such as livestock, companion animals or humans with the clinical breakpoint concentration of some of the tested antimicrobials.

Given that AMR is common in birds, it can act as a contaminant for the environment with indiscriminate defecation from birds^35^. Assessment of environmental contamination and AMR is difficult to quantify, largely due to different soil ecosystems showing different AMR carriage levels, with Osbiston et al.^36^ showing that land use (farming vs recreational for example) can alter the levels of AMR and the resistance profiles of the bacteria found. The impact which bird faecal matter has on this is unknown.

### *Salmonella*

Comparison of our results with those of previous studies is complicated by the high diversity of bird species, and a large-scale comparison of each individual species would be beneficial to allow for a more meaningful comparison. Many previous studies are carried out in association with farms, potentially to quantify the risk of introduction of the focal pathogen; however, given the routinely high levels of AMR bacteria and *Salmonella* isolated from many farmed livestock species, the results from these previous studies may be highly skewed^37^. In addition, rather than the methods of sampling using mist nets as employed in this study, many focus on birds in rescue shelters, birds such as pigeons that have been culled, or specific focal species such as raptors or large animals such as storks; thus these studies often include only small numbers of passerine birds despite their tendency to be more numerous and widespread. Species with high *Salmonella* prevalence in this study included the Eurasian blackcap (*Sylvia atricapilla),* Eurasian blue tits (*Cyanistes caeruleus*) and house sparrows (*Passer domesticus*). Unfortunately, few previous studies target these species, with a few studies reporting *Salmonella* spp. in the Eurasian blackcap (prevalence of 1/8^38^; two positives for *Salmonella* spp. but does not say how many blackcaps were tested specifically^39^)*,* the Eurasian blue tit (one positive from six samples tested^38^; 3/14 positive samples^40^; 4 of 36 eggs^41^) and house sparrows (7/31 positives^40^; 7/11 carcasses^42^; 33/50 infected with *Salmonella* spp.^43^). Other studies have suggested that species such as house sparrows do not shed much *Salmonella* Typhimurium^44^.

This is particularly surprising given that studies report that wild bird salmonellosis can pose a risk to human and animal health, and given that a large number of UK homeowners provide supplementary food to garden birds^6^, this offers a simple yet successful method for introduction of *Salmonella* into the human environment and potentially for infection^45,46^. Indeed, previous studies have linked wild songbirds to *Salmonella* outbreaks in humans^47^.

That said, our findings (11.6% *Salmonella* spp. positivity) are in line with, or slightly higher than, other studies, including a reported 6.4% on average for carriage in wild birds in Poland^48^, but this varied dramatically between bird species, with the Eurasian siskin (*Carduelis spinus*) and the greenfinch (*Carduelis chloris*) being around 33% (based on 30 or more samples). In addition, a prevalence of 12.3% *Salmonella* spp. carriage has been reported from birds in Spain^49^. A similar prevalence (7.4%) was observed in a study in Croatia^50^ and lower prevalences were observed in South Korea (0.93%^51^) and on the Austria- Czech Republic border (2.2%^52^). However, higher prevalences have also been found: for example, in Texas, USA, 17% of wild birds were positive for *Salmonella* spp.^53^, as were 13.5% of birds tested in Bangladesh^54^.

The isolation of *S.* Typhimurium is relatively common among various bird species^10,43,55,56^, which concurs with our findings. Many of the other strains have also been isolated in previous studies^37,40,57–61^. Interestingly, some of these strains have been isolated from other animals including dogs^62^ and livestock^63,64^, suggesting that there may be transmission either from birds to these animals, or vice versa^65,66^. Multi-drug resistance has been reported commonly within *Salmonella* isolates, some of which were from wild birds. Multi drug resistance poses problems for treatment opportunities in infected animals and humans, and thus the clinical shedding of these bacteria is of high importance. Indeed, *Salmonella* spp. isolated from wild birds are commonly MDR, for example 86.7% of 15 isolates obtained by Martín-Maldonado et al.^49^.

High levels of tetracycline resistance have been reported previously^67^, with chloramphenicol and ampicillin resistance equally commonly seen in wild bird *Salmonella* isolates^68,69^. The lack of resistance to nalidixic acid seen within this study differs from others which report high levels of resistance^49,67,70^, although the reasons for this are unclear.

Risk factor analyses for the carriage of *Salmonella* spp. in wild birds are lacking, as most studies focus on specific populations, such as those admitted to rescue hospitals, or single species. Younger birds have been found as more likely to be positive for *Salmonella* than older birds in a range of species^49^, although we did not find any difference between ages in our study. However, we did find that all intercontinental migrants carrying *Salmonella* were carrying MDR *Salmonella*, which is particularly interesting as these species tend to be reliant on invertebrate food rather than food provided by householders. The review by Blazar, Allard, and Lienau^71^ suggested that a wide variety of different insects which could act as prey for passerine birds, such as lesser mealworm, *Alphitobius diaperinus* (Panzer) can carry several pathogens, including *Salmonella* spp. or *E. coli* and act as successful vectors, suggesting a potential transmission route. Similarly, dipteran flies, commonly eaten by a range of bird species including migrants, can also vector *Salmonella* spp.^72^.

### *Campylobacter*

We found *Campylobacter* spp. presence to differ between species, and many studies have found similar results. However, many focus specifically on *C. jejuni* because this is the most common species to cause disease in humans^73^. Indeed, it has been reported that *C. jejuni* from wild birds are a consistent cause of human disease^9^. Mencía-Gutierrez et al.^20^ report a prevalence of *Campylobacter* spp. in 7.5% of raptors from Spain, with *C. jejuni* making up 88.5% of the isolates, and Waldenström et al.^74^ report a prevalence of 21.6% in Sweden, but this was highly variable across different species, and 24.8% prevalence was reported in Italy at a wildlife rescue centre with 94.23% of these being *C. jejuni* and the remained being *C. coli*^75^. Similar to the prevalence obtained here, 15.3% was reported in South Korea^76^ and ranged from 8.5% to 50% depending on the bird species in Antarctic and sub-Antarctic regions^77^. Many of these studies target different bird species, in different areas, and in some cases use different laboratory methodologies, and as such, the results are difficult to compare.

The specific *Campylobacter* species which we isolated are very much in line with other studies, although the most common species seems to be variable. Similar to our study, *C. jejuni* was most common in wild birds associated with a Danish livestock farm^78^, in an Italian rescue shelter^75^, in the mid-Atlantic region of the USA^79,80^, in Northern Poland^81^ and from wild birds of prey in Spain^20^. By contrast, *C. lari* was most commonly found in Sweden^74^, and in the Antarctic peninsula^77^, although we found only a slightly lower prevalence of *C. lari* compared to *C. jejuni*.

Drug resistance levels appear to vary widely across studies, dependent upon area and bird species tested. Variation among the laboratory protocols used also makes direct comparisons difficult. Resistance to tetracycline is common in many studies, and has been reported previously^75,82^, but tetracycline and amoxicillin resistance was lower^83,84^. Erythromycin resistance is variable, with low levels reported by Casalino et al.^75^ and Du et al.^82^ whereas Casalino et al.^75^ also reported high levels of resistance to trimethoprim-sulfamethoxazole. By contrast, no drug resistance was found to erythromycin and low resistance was found to amoxicillin by Waldenström et al.^74^ and Kürekci et al.^85^ found no resistance to erythromycin or tetracycline.

We found juvenile birds to be more than twice as likely to be infected by *Campylobacter* spp. than adults, in agreement with Taff et al.^86^, although other studies find no association^20,87^. With regards to risk factors from the environment, farmland and animals have been shown to be risk factors for an increased level of *Campylobacter* spp. carriage in wild birds^78^ and it has been suggested that wild birds may play a role in infection of livestock with *Campylobacter* spp.^88^ although other studies suggest that the converse is true^89^.

Bird species associated with human habitation had a higher prevalence of *Campylobacter* spp. than those not associated with human habituation, which poses many potential questions, including whether the pathogen comes from human food, or possibly from contact with bird feeders. It also increases the potential risk of transmission from bird to human (or vice versa); indeed, previous epidemics have been linked to wild bird contact^90^. The risk factors for carriage of *Campylobacter* spp., and the risks which they pose to human health, require further research.

### *Enterococcus*

Previous studies suggest that the prevalence of *Enterococcus* varies dramatically depending on the bird species tested, geographical area, and the laboratory methodologies used^91^. However, isolates of *Enterococcus* found in wild birds can cause infections in humans^92^.

The prevalence of *Enterococcus* obtained in this study is similar, if slightly higher than that reported in other studies, including 63.3% reported in the Azores archipelago^93^, 65.8% in Tunisia^94^, 66.7% in Poland^95^ and 74% in Slovakia^96^. The species isolated in this study are similar to those isolated in other studies, with *E. faecium* being most common in many studies^93–95,97–99^. The other species were isolated in lower numbers in various studies which is also in line with our findings^93–95,97,99^.

Drug resistance is commonly seen in *Enterococcus* spp., with nearly every isolate obtained by Cagnoli et al.^100^ being described as MDR, and this bacterium has become a common indicator of environmental contamination with faecal matter due to its ability to rapidly uptake antimicrobial resistance genes^101^. Resistance to vancomycin is common, especially within *E. faecium* and *E. faecalis* isolates^102^. Tetracycline resistance was also common in *Enterococcus* isolates in this study, and this is also commonly seen in farm animal isolated *Enterococcus*^103^ which may offer a potential transmission route for the bacteria, although the direction is unknown. In addition, other studies have reported high levels of resistance of *Enterococcus* to tetracycline^93,95,98,104^. Surprisingly in this study, resistance to teicoplanin and streptomycin were low, which is in contrast to the results reported by Dec et al.^104^, although other studies support the low resistance finding to these antimicrobials^93,95,98^. Previous studies have suggested that the level of antimicrobial resistance genes is not consistent across the year, with crows shown to carry lower levels of antimicrobial resistance in the summer compared to ducks and gulls, and the authors attribute this to seasonal variation in food resources due to winter foraging in waste disposal areas and highly populated areas compared to summer where seeds and grain make up more of the diets^105,106^. This concurs with our findings of a decline in the prevalence of MDR *Enterococcus* through the season: it is likely that temperature, humidity and density of animals will have an impact on the carriage and transmission of antimicrobial resistance genes^107^.

Birds with a wider dietary range may tend to carry more pathogens^98,108^, which concurs with our finding of a tendency for insectivorous birds to carry a higher prevalence of *Enterococcus* than other birds. Insects have been shown to be a common carrier of *Enterococcus* spp. as well as other bacteria, and this may allow for a route of transmission to birds^109^. In addition, *Enterococcus* and other antimicrobial resistant bacteria have been isolated from caterpillars, which act as one of the major food sources for many insectivorous birds and may allow for a transmission route^110^.

Whilst the prevalence of *Enterococcus* spp. within birds in this study is high, this does not mean that all of these isolates maybe pathogenic, as *Enterococcus* spp. is a widely used probiotic for benefits of digestion^111^. Whilst all *Enterococcus* spp. carrying AMR genes can lead to horizontal gene transfer to other bacterial species^112^, to further understand the pathogenic nature of the isolates would involve some analysis of virulence genes^113^, and other phenotypic differences such as ability to withstand the low pH of the GI tract^114^.

### *E. coli*

*E. coli* is very commonly isolated from the faecal samples of many animals and is often used to assess antimicrobial resistance. Consequently, many studies have been carried out in birds, but results depend on the geographic areas and the bird species tested. This is epitomised by the study by Stedt et al.^115^ who reported a variation in *E. coli* resistance in gulls in Europe varying from 61.2% in Spain to 8.3% in Denmark. In the Azores archipelago, AMR within *E. coli* isolates was shown to be 24.3%^93^, but increases to values of 63% in Turkey^116^. Resistance levels vary, although similarly to our study, resistance to ampicillin tends to be common^24,25,93,117,118^, although other studies such as that conducted in Poland^119^ report a lower prevalence at 28.1%, and 16.7%^120^. Total resistance to ampicillin has also been reported^121^. In addition, nalidixic acid resistance also seems common in wild birds in various parts of the world^24,118,122^. However, the resistance to amikacin seems variable with some studies reporting a low level of resistance to this antimicrobial^93,120^, or in some cases, no resistance at all^24,117,123^ whereas Prandi et al.^124^ report amikacin resistance of 17.9%. Kanamycin resistance again varies among wild bird *E. coli* isolates, with 18.7% resistance reported by Nowaczek et al*.*^119^ but much higher resistance of 38% observed in birds in Turkey^90^.

Levels of MDR also tend to vary, with 39.6% of *E. coli* isolates being resistant to three or more antimicrobials in Italy^124^, 31.2% MDR observed in Poland^119^, 38% in Brazil^125^, 33.5% in Lithuania^117^ and 38.6% in Poland^118^. Similar to this study, Yuan et al.^120^ found 61.9% of 118 isolates which were classed as MDR. Total MDR (i.e. 100% of isolated bacteria) was observed in some villages in Malaysia, but the levels varied by area^126^. Interestingly, our findings suggested a higher diet diversity in individuals carrying MDR *E. coli* compared to those carrying non-MDR *E. coli*, suggesting that exposure to *E. coli* in multiple food types may increase the likelihood of MDR^98^.

## Conclusion

The high level of pathogen carriage observed within this study from birds within the UK acts as a timely reminder of the risks which bird contact and bird faecal matter may pose, and the impacts that land management can have on wildlife. Although contact with wild birds is generally limited, risks may be posed from bird feeders, or through indiscriminate defecation in urban or suburban areas leading to environmental contamination. This in turn may lead to infections of other animals such as companion animals or livestock and could potentially enter the food chain leading to zoonotic risks. Although not tested in this study, the presence of antimicrobial resistance genes is also likely to pose potential risks to humans and animals. It is crucial that further research tests potential mechanisms of reducing levels of MDR bacteria in wildlife, potentially through increased hygiene of supplementary food resources.

## Electronic supplementary material

Below is the link to the electronic supplementary material.

Supplementary Material 1

## Data Availability

Data are available through FigShare at the following DOIs. Analysis code is available at 10.6084/m9.figshare.26160301, with the full dataset available at 10.6084/m9.figshare.26160361, the full dataset excluding species with n < 4 available at 10.6084/m9.figshare.26160343, all Campylobacter positive samples available at 10.6084/m9.figshare.26160334, Campylobacter positive samples excluding host species with n < 4 available at 10.6084/m9.figshare.26160349, all Salmonella positive samples available at 10.6084/m9.figshare.26160352, and all Enterococcus positive samples available at 10.6084/m9.figshare.26160358.
