# Supplementary material for: High prevalence of multi-drug-resistant bacteria in faecal samples from UK passerine birds
Source: Sci Rep. 2025 Aug 1;15:28130. doi: 10.1038/s41598-025-13012-4 (PMC12316977; doi:10.1038/s41598-025-13012-4)
Supplement: Supplementary file 1 — Supplementary Material 1 [file 41598_2025_13012_MOESM1_ESM.docx]

**Supplementary data**

Table S1. Primers used for the PCR assays within the study, including product size (bp) and gene targets

| **Bacterial genus** | **Bacteria species** | **Primer name** | **Product size (bp)** | **Sequence (5'-3')** | **Gene target** |
| --- | --- | --- | --- | --- | --- |
| *Campylobacter* spp. | *C. coli* | CCF | 126 | GTAAAACCAAAGCTTATCGTG | *C. coli glyA* |
|  |  | CCR |  | TCCAGCAATGTGTGCAATG |  |
|  | *C. lari* | CLF | 251 | TAGAGAGATAGCAAAAGAGA | *C. lari glyA* |
|  |  | CLR |  | TACACATAATAATCCCACCC |  |
|  | *C. upsaliensis* | CUF | 204 | AATTGAAACTCTTGCTATCC | *C. upsaliensis glyA* |
|  |  | CUR |  | TCATACATTTTACCCGAGCT |  |
|  | *C. jejuni* | 23SF | 650 | TATACCGGTAAGGAGTGCTGGAG | *C. jejuni* 23S rRNA |
|  |  | 23SR |  | ATCAATTAACCTTCGAGCACCG |  |
| *E. coli* | | ECP79F | 503 | GAAGCTTGCTTCTTTGCT | *E. coli* 16s rRNA |
|  |  | ECR620R |  | GAGCCCGGGGATTTCACAT |  |
| Salmonella spp. | | invA_176F | 116 | CAACGTTTCCTGCGGTACTGT | *Salmonella invA* |
|  |  | invA_291R |  | CCCGAACGTGGCGATAATT |  |
| *Enterococcus* spp. | *Enterococcus* spp. | Ent1 |  | 5′-TACTGACAAACCATTCATGATG-3′ | *Enterococcus tuf* |
|  |  | Ent2 |  | 5′-AACTTCGTCACCAACGCGAAC-3′ |  |
|  | *E. durans* | DU1 | 295 | CCTACTGATATTAAGACAGCG | *E. durans sodA* |
|  |  | DU2 |  | TAATCCTAAGATAGGTGTTTG |  |
|  | *E. faecalis* | FL1 | 360 | ACTTATGTGACTAACTTAACC | *E. faecalis sodA* |
|  |  | FL2 |  | TAATGGTGAATCTTGGTTTGG |  |
|  | *E. faecium* | FM1 | 215 | GAAAAAACAATAGAAGAATTAT | *E. faecium sodA* |
|  |  | FM2 |  | TGCTTTTTTGAATTCTTCTTTA |  |
|  | *E. hirae* | HI1 | 187 | CTTTCTGATATGGATGCTGTC | *E. hirae sodA* |
|  |  | HI2 |  | TAAATTCTTCCTTAAATGTTG |  |

Table S2. Antibiotic susceptibility of *Salmonella* isolates recovered from passerine faecal samples, split by host species.

| Species (no. positive) | AK | AMC | AMP | CRO | C | CN | NA | NX | SXT | TE |
| --- | --- | --- | --- | --- | --- | --- | --- | --- | --- | --- |
| Blackbird (n=2) | | |  |  |  |  |  |  |  |  |
| Resistant | 0 | 0 | 0 | 0 | 2 | 0 | 0 | 0 | 0 | 1 |
| Intermediate | 0 | 0 | 0 | 1 | 0 | 0 | 0 | 1 | 1 | 1 |
| Susceptible | 2 | 2 | 2 | 1 | 0 | 2 | 2 | 1 | 1 | 0 |
| Blackcap (n=5) |  |  |  |  |  |  |  |  |  |  |
| Resistant | 0 | 2 | 3 | 4 | 3 | 0 | 0 | 0 | 3 | 5 |
| Intermediate | 0 | 1 | 0 | 0 | 0 | 2 | 1 | 0 | 1 | 0 |
| Susceptible | 5 | 2 | 2 | 1 | 2 | 3 | 4 | 5 | 1 | 0 |
| Blue tit (n=3) |  |  |  |  |  |  |  |  |  |  |
| Resistant | 0 | 0 | 2 | 1 | 1 | 0 | 0 | 0 | 1 | 1 |
| Intermediate | 0 | 1 | 0 | 1 | 0 | 0 | 0 | 0 | 0 | 1 |
| Susceptible | 3 | 2 | 1 | 1 | 2 | 3 | 3 | 3 | 2 | 1 |
| Chaffinch (n=2) |  |  |  |  |  |  |  |  |  |  |
| Resistant | 0 | 1 | 0 | 0 | 0 | 0 | 0 | 0 | 1 | 2 |
| Intermediate | 0 | 0 | 0 | 0 | 1 | 0 | 0 | 0 | 1 | 0 |
| Susceptible | 2 | 1 | 2 | 2 | 1 | 2 | 2 | 2 | 0 | 0 |
| Chiffchaff (n=1) |  |  |  |  |  |  |  |  |  |  |
| Resistant | 0 | 1 | 0 | 1 | 1 | 0 | 0 | 0 | 0 | 1 |
| Intermediate | 0 | 0 | 0 | 0 | 0 | 0 | 0 | 0 | 1 | 0 |
| Susceptible | 1 | 0 | 1 | 0 | 0 | 1 | 1 | 1 | 0 | 0 |
| Dunnock (n=2) |  |  |  |  |  |  |  |  |  |  |
| Resistant | 0 | 1 | 1 | 1 | 0 | 0 | 0 | 0 | 1 | 1 |
| Intermediate | 0 | 0 | 0 | 1 | 0 | 0 | 1 | 0 | 0 | 0 |
| Susceptible | 2 | 1 | 1 | 0 | 2 | 2 | 1 | 2 | 1 | 1 |
| Goldfinch (n=2) |  |  |  |  |  |  |  |  |  |  |
| Resistant | 0 | 0 | 2 | 1 | 1 | 0 | 0 | 0 | 1 | 1 |
| Intermediate | 0 | 0 | 0 | 1 | 0 | 0 | 0 | 0 | 0 | 1 |
| Susceptible | 2 | 2 | 0 | 0 | 1 | 2 | 2 | 2 | 1 | 0 |
| House sparrow (n=8) |  |  |  |  |  |  |  |  |  |  |
| Resistant | 1 | 3 | 4 | 4 | 6 | 0 | 1 | 0 | 4 | 5 |
| Intermediate | 0 | 0 | 0 | 2 | 1 | 2 | 0 | 2 | 2 | 0 |
| Susceptible | 7 | 5 | 4 | 2 | 1 | 6 | 7 | 6 | 2 | 3 |
| Robin (n=2) |  |  |  |  |  |  |  |  |  |  |
| Resistant | 0 | 0 | 0 | 0 | 2 | 0 | 0 | 0 | 0 | 2 |
| Intermediate | 0 | 0 | 0 | 2 | 0 | 0 | 0 | 0 | 0 | 0 |
| Susceptible | 2 | 2 | 2 | 0 | 0 | 2 | 2 | 2 | 2 | 0 |
| Whitethroat (n=1) |  |  |  |  |  |  |  |  |  |  |
| Resistant | 0 | 1 | 1 | 1 | 1 | 0 | 0 | 0 | 0 | 0 |
| Intermediate | 0 | 0 | 0 | 0 | 0 | 0 | 0 | 0 | 0 | 0 |
| Susceptible | 1 | 0 | 0 | 0 | 0 | 1 | 1 | 1 | 1 | 1 |
| Wren (n=2) |  |  |  |  |  |  |  |  |  |  |
| Resistant | 0 | 1 | 2 | 1 | 0 | 1 | 0 | 0 | 2 | 2 |
| Intermediate | 0 | 0 | 0 | 0 | 0 | 0 | 1 | 0 | 0 | 0 |
| Susceptible | 2 | 1 | 0 | 1 | 2 | 1 | 1 | 2 | 0 | 0 |
| Total (n=30) |  |  |  |  |  |  |  |  |  |  |
| Resistant | 1 | 10 | 15 | 14 | 17 | 1 | 1 | 0 | 13 | 21 |
| Intermediate | 0 | 2 | 0 | 8 | 2 | 4 | 3 | 3 | 6 | 3 |
| Susceptible | 29 | 18 | 15 | 8 | 11 | 25 | 26 | 27 | 11 | 6 |

AK: Amikacin (30 mg/ml); AMC: Amoxicillin-Clavulanic acid (20 mg/ml); AMP: Ampicillin (10 mg/ml); CRO: Ceftriaxone (30 mg); C: Chloramphenicol (30 mg/ml); CN: Gentamicin (10 mg/ml); NA: Nalidixic acid (30 mg/ml); NX: Norfloxacin (10 mg/ml); SXT: Sulfamethoxazole-trimethoprim (25 mg/ml); TE: Tetracycline (30 mg/ml)

Table S3. Antibiotic susceptibility of *Campylobacter* isolates recovered from passerine faecal samples.

| Species (no. positive) | AZM | AMX | C | CIP | CN | ENR | E | NA | SXT | TE |
| --- | --- | --- | --- | --- | --- | --- | --- | --- | --- | --- |
| Blackbird (n=3) | | |  |  |  |  |  |  |  |  |
| Resistant | 3 | 1 | 2 | 2 | 2 | 0 | 1 | 0 | 0 | 1 |
| Intermediate | 0 | 1 | 0 | 1 | 0 | 0 | 0 | 2 | 0 | 1 |
| Susceptible | 0 | 1 | 1 | 0 | 1 | 3 | 2 | 1 | 3 | 1 |
| Blackcap (n=3) |  |  |  |  |  |  |  |  |  |  |
| Resistant | 2 | 1 | 0 | 2 | 1 | 0 | 2 | 1 | 0 | 2 |
| Intermediate | 0 | 1 | 1 | 1 | 0 | 0 | 0 | 1 | 1 | 1 |
| Susceptible | 1 | 1 | 2 | 0 | 2 | 3 | 1 | 1 | 2 | 0 |
| Blue tit (n=5) |  |  |  |  |  |  |  |  |  |  |
| Resistant | 2 | 2 | 0 | 3 | 4 | 2 | 2 | 5 | 1 | 3 |
| Intermediate | 1 | 2 | 2 | 1 | 0 | 2 | 1 | 0 | 0 | 0 |
| Susceptible | 2 | 1 | 3 | 1 | 1 | 1 | 2 | 0 | 4 | 2 |
| Chaffinch (n=3) |  |  |  |  |  |  |  |  |  |  |
| Resistant | 3 | 3 | 0 | 2 | 1 | 0 | 3 | 0 | 0 | 3 |
| Intermediate | 0 | 0 | 1 | 0 | 1 | 0 | 0 | 1 | 1 | 0 |
| Susceptible | 0 | 0 | 2 | 1 | 1 | 3 | 0 | 2 | 2 | 0 |
| Chiffchaff (n=1) |  |  |  |  |  |  |  |  |  |  |
| Resistant | 1 | 0 | 1 | 0 | 0 | 0 | 0 | 1 | 0 | 1 |
| Intermediate | 0 | 0 | 0 | 0 | 0 | 0 | 0 | 0 | 0 | 0 |
| Susceptible | 0 | 1 | 0 | 1 | 1 | 1 | 1 | 0 | 1 | 0 |
| Goldfinch (n=1) |  |  |  |  |  |  |  |  |  |  |
| Resistant | 1 | 1 | 1 | 0 | 0 | 0 | 1 | 0 | 0 | 1 |
| Intermediate | 0 | 0 | 0 | 0 | 0 | 0 | 0 | 0 | 0 | 0 |
| Susceptible | 0 | 0 | 0 | 1 | 1 | 1 | 0 | 1 | 1 | 0 |
| Great tit (n=1) |  |  |  |  |  |  |  |  |  |  |
| Resistant | 1 | 1 | 0 | 0 | 0 | 1 | 1 | 0 | 0 | 0 |
| Intermediate | 0 | 0 | 0 | 0 | 1 | 0 | 0 | 0 | 0 | 0 |
| Susceptible | 0 | 0 | 1 | 1 | 0 | 0 | 0 | 1 | 1 | 1 |
| Greenfinch (n=1) |  |  |  |  |  |  |  |  |  |  |
| Resistant | 1 | 1 | 0 | 1 | 0 | 0 | 1 | 0 | 0 | 1 |
| Intermediate | 0 | 0 | 0 | 0 | 0 | 0 | 0 | 0 | 0 | 0 |
| Susceptible | 0 | 0 | 1 | 0 | 1 | 1 | 0 | 1 | 1 | 0 |
| House sparrow (n=19) |  |  |  |  |  |  |  |  |  |  |
| Resistant | 5 | 12 | 4 | 5 | 8 | 3 | 10 | 1 | 4 | 12 |
| Intermediate | 6 | 4 | 1 | 2 | 3 | 4 | 3 | 8 | 5 | 6 |
| Susceptible | 8 | 3 | 14 | 12 | 8 | 12 | 6 | 10 | 10 | 1 |
| Reed warbler (n=1) |  |  |  |  |  |  |  |  |  |  |
| Resistant | 1 | 1 | 0 | 0 | 1 | 1 | 1 | 0 | 0 | 0 |
| Intermediate | 0 | 0 | 0 | 0 | 0 | 0 | 0 | 1 | 0 | 0 |
| Susceptible | 0 | 0 | 1 | 1 | 0 | 0 | 0 | 0 | 1 | 1 |
| Robin (n=4) |  |  |  |  |  |  |  |  |  |  |
| Resistant | 0 | 1 | 1 | 2 | 2 | 2 | 1 | 3 | 1 | 2 |
| Intermediate | 1 | 2 | 1 | 2 | 1 | 1 | 1 | 0 | 0 | 0 |
| Susceptible | 3 | 1 | 2 | 0 | 1 | 1 | 2 | 1 | 3 | 2 |
| Song thrush (n=1) |  |  |  |  |  |  |  |  |  |  |
| Resistant | 0 | 1 | 0 | 0 | 0 | 0 | 1 | 0 | 0 | 1 |
| Intermediate | 0 | 0 | 0 | 0 | 1 | 0 | 0 | 0 | 0 | 0 |
| Susceptible | 1 | 0 | 1 | 1 | 0 | 1 | 0 | 1 | 1 | 0 |
| Whitethroat (n=3) |  |  |  |  |  |  |  |  |  |  |
| Resistant | 0 | 2 | 2 | 2 | 1 | 0 | 2 | 1 | 1 | 1 |
| Intermediate | 0 | 1 | 1 | 1 | 1 | 1 | 0 | 1 | 0 | 2 |
| Susceptible | 3 | 0 | 0 | 0 | 1 | 2 | 1 | 1 | 2 | 0 |
| Willow warbler (n=1) |  |  |  |  |  |  |  |  |  |  |
| Resistant | 0 | 1 | 0 | 1 | 0 | 0 | 1 | 0 | 0 | 0 |
| Intermediate | 0 | 0 | 0 | 0 | 1 | 0 | 0 | 1 | 0 | 0 |
| Susceptible | 1 | 0 | 1 | 0 | 0 | 1 | 0 | 0 | 1 | 1 |
| Wren (n=2) |  |  |  |  |  |  |  |  |  |  |
| Resistant | 1 | 2 | 0 | 2 | 1 | 0 | 2 | 0 | 0 | 1 |
| Intermediate | 1 | 0 | 0 | 0 | 0 | 0 | 0 | 0 | 1 | 1 |
| Susceptible | 0 | 0 | 2 | 0 | 1 | 2 | 0 | 2 | 1 | 0 |
| Total (n=49) |  |  |  |  |  |  |  |  |  |  |
| Resistant | 21 | 30 | 11 | 22 | 21 | 9 | 29 | 12 | 7 | 29 |
| Intermediate | 9 | 11 | 7 | 8 | 9 | 8 | 5 | 15 | 8 | 11 |
| Susceptible | 19 | 8 | 31 | 19 | 19 | 32 | 15 | 22 | 34 | 9 |

AZM: Azithromycin (15 mg); AMX: Amoxicillin (30 mg); C: Chloramphenicol (30 mg); CIP: Ciprofloxacin (5 mg); CN: Gentamicin (10 mg); ENR: Enrofloxacin (5 mg); E: Erythromycin (15 mg); NA: Nalidixic acid (30 mg); SXT: Trimethoprim-sulfamethoxazole (25 mg); TE: Tetracycline 173 (30 mg)

Table S4. Antibiotic susceptibility of *Enterococcus* isolates recovered from passerine faecal samples.

| Species (no. positive) | AMP | C | CIP | CN | E | K | S | Ti | TE | VA |
| --- | --- | --- | --- | --- | --- | --- | --- | --- | --- | --- |
| Blackbird (n=3) | | |  |  |  |  |  |  |  |  |
| Resistant | 0 | 2 | 2 | 1 | 1 | 1 | 0 | 0 | 1 | 3 |
| Susceptible | 3 | 1 | 1 | 2 | 2 | 2 | 3 | 3 | 2 | 0 |
| Blackcap (n=19) | AMP | C | CIP | CN | E | K | S | Ti | TE | VA |
| Resistant | 5 | 3 | 5 | 3 | 7 | 3 | 2 | 1 | 3 | 8 |
| Susceptible | 14 | 16 | 14 | 16 | 12 | 16 | 17 | 18 | 16 | 11 |
| Blue tit (n=24) | AMP | C | CIP | CN | E | K | S | Ti | TE | VA |
| Resistant | 3 | 7 | 8 | 2 | 8 | 9 | 3 | 1 | 8 | 11 |
| Susceptible | 21 | 17 | 16 | 22 | 16 | 15 | 21 | 23 | 16 | 13 |
| Bullfinch (n=3) | AMP | C | CIP | CN | E | K | S | Ti | TE | VA |
| Resistant | 0 | 0 | 0 | 0 | 0 | 0 | 0 | 0 | 0 | 0 |
| Susceptible | 3 | 3 | 3 | 3 | 3 | 3 | 3 | 3 | 3 | 3 |
| Chaffinch (n=24) | AMP | C | CIP | CN | E | K | S | Ti | TE | VA |
| Resistant | 3 | 3 | 5 | 2 | 5 | 5 | 2 | 1 | 6 | 8 |
| Susceptible | 21 | 21 | 19 | 22 | 19 | 19 | 22 | 23 | 18 | 16 |
| Chiffchaff (n=2) | AMP | C | CIP | CN | E | K | S | Ti | TE | VA |
| Resistant | 0 | 0 | 0 | 0 | 0 | 0 | 0 | 0 | 0 | 1 |
| Susceptible | 3 | 3 | 3 | 3 | 3 | 3 | 3 | 3 | 3 | 2 |
| Dunnock (n=12) | AMP | C | CIP | CN | E | K | S | Ti | TE | VA |
| Resistant | 1 | 3 | 5 | 1 | 4 | 4 | 0 | 1 | 7 | 3 |
| Susceptible | 11 | 9 | 7 | 11 | 8 | 8 | 12 | 11 | 5 | 9 |
| Goldfinch (n=3) | AMP | C | CIP | CN | E | K | S | Ti | TE | VA |
| Resistant | 0 | 1 | 1 | 0 | 0 | 2 | 1 | 0 | 1 | 1 |
| Susceptible | 3 | 2 | 1 | 3 | 1 | 1 | 3 | 3 | 2 | 2 |
| Great tit (n=8) | AMP | C | CIP | CN | E | K | S | Ti | TE | VA |
| Resistant | 2 | 1 | 2 | 1 | 2 | 1 | 0 | 1 | 2 | 3 |
| Susceptible | 6 | 7 | 6 | 7 | 6 | 7 | 8 | 7 | 6 | 5 |
| Greenfinch (n=4) | AMP | C | CIP | CN | E | K | S | Ti | TE | VA |
| Resistant | 3 | 0 | 1 | 1 | 0 | 0 | 0 | 0 | 1 | 0 |
| Susceptible | 1 | 4 | 3 | 3 | 4 | 4 | 4 | 4 | 3 | 4 |
| House sparrow (n=31) | AMP | C | CIP | CN | E | K | S | Ti | TE | VA |
| Resistant | 6 | 6 | 7 | 5 | 9 | 11 | 3 | 2 | 14 | 10 |
| Susceptible | 25 | 25 | 24 | 26 | 22 | 20 | 28 | 29 | 17 | 21 |
| Lesser whitethroat (n=1) | AMP | C | CIP | CN | E | K | S | Ti | TE | VA |
| Resistant | 1 | 0 | 0 | 1 | 0 | 1 | 0 | 0 | 0 | 0 |
| Susceptible | 0 | 1 | 1 | 0 | 1 | 0 | 1 | 1 | 1 | 1 |
| Long-tailed tit (n=2) | AMP | C | CIP | CN | E | K | S | Ti | TE | VA |
| Resistant | 0 | 0 | 1 | 1 | 0 | 1 | 1 | 0 | 1 | 1 |
| Susceptible | 2 | 2 | 1 | 1 | 2 | 1 | 1 | 2 | 1 | 1 |
| Reed bunting (n=5) | AMP | C | CIP | CN | E | K | S | Ti | TE | VA |
| Resistant | 0 | 1 | 1 | 0 | 1 | 1 | 0 | 0 | 2 | 2 |
| Susceptible | 5 | 4 | 4 | 5 | 4 | 4 | 5 | 4 | 3 | 3 |
| Reed warbler (n=6) | AMP | C | CIP | CN | E | K | S | Ti | TE | VA |
| Resistant | 1 | 1 | 1 | 1 | 1 | 2 | 1 | 0 | 2 | 1 |
| Susceptible | 5 | 5 | 5 | 5 | 5 | 4 | 5 | 6 | 4 | 5 |
| Robin (n=19) | AMP | C | CIP | CN | E | K | S | Ti | TE | VA |
| Resistant | 5 | 5 | 4 | 0 | 2 | 2 | 0 | 0 | 5 | 7 |
| Susceptible | 14 | 14 | 15 | 19 | 17 | 17 | 19 | 19 | 14 | 12 |
| Sedge warbler (n=2) | AMP | C | CIP | CN | E | K | S | Ti | TE | VA |
| Resistant | 0 | 0 | 0 | 0 | 0 | 0 | 0 | 0 | 0 | 1 |
| Susceptible | 2 | 2 | 2 | 2 | 2 | 2 | 2 | 2 | 2 | 1 |
| Song thrush (n=1) | AMP | C | CIP | CN | E | K | S | Ti | TE | VA |
| Resistant | 0 | 0 | 0 | 0 | 0 | 0 | 0 | 0 | 0 | 0 |
| Susceptible | 1 | 1 | 1 | 1 | 1 | 1 | 1 | 1 | 1 | 1 |
| Whitethroat (n=13) | AMP | C | CIP | CN | E | K | S | Ti | TE | VA |
| Resistant | 0 | 4 | 3 | 2 | 4 | 3 | 0 | 1 | 5 | 4 |
| Susceptible | 13 | 9 | 10 | 11 | 9 | 10 | 13 | 12 | 8 | 9 |
| Willow warbler (n=3) | AMP | C | CIP | CN | E | K | S | Ti | TE | VA |
| Resistant | 0 | 0 | 0 | 0 | 0 | 0 | 0 | 0 | 0 | 0 |
| Susceptible | 3 | 3 | 3 | 3 | 3 | 3 | 3 | 3 | 3 | 3 |
| Wren (n=14) | AMP | C | CIP | CN | E | K | S | Ti | TE | VA |
| Resistant | 3 | 2 | 4 | 2 | 3 | 2 | 0 | 1 | 4 | 2 |
| Susceptible | 11 | 12 | 10 | 12 | 11 | 12 | 14 | 13 | 10 | 12 |
| Yellowhammer (n=4) | AMP | C | CIP | CN | E | K | S | Ti | TE | VA |
| Resistant | 0 | 0 | 0 | 1 | 0 | 0 | 1 | 1 | 1 | 2 |
| Susceptible | 4 | 4 | 4 | 3 | 4 | 4 | 3 | 3 | 3 | 2 |
| Total (n=203) | AMP | C | CIP | CN | E | K | S | Ti | TE | VA |
| Resistant | 33 | 39 | 50 | 24 | 47 | 48 | 14 | 10 | 63 | 68 |
| Susceptible | 170 | 164 | 153 | 179 | 156 | 155 | 189 | 193 | 140 | 135 |

AMP: Ampicillin (10 mg/ml); C: Chloramphenicol (30 mg/ml); CIP: Ciprofloxacin (5 mg/ml); CN: Gentamicin (120 mg/ml); E: Erythomycin (15 mg/ml); K: Kanamycin (120 mg/ml); S: Streptomycin (300 mg/ml); Ti: Teicoplanin (30 mg/ml); TE: Tetracycline (30 mg/ml); VA: Vancomycin (30 mg/ml)

Table S5. Antibiotic susceptibility of *E. coli* isolates recovered from passerine faecal samples. The three numbers in each cell represent three replicates; total data are the mean ± SE of the three replicate totals.

| Species (no. positive) | AK | AMC | AMP | AZ | CTX | FOX | CAZ | CFT | CRO | C | CIP | ENR | E | CN | IMP | K | M | NA | S | TE | TI | SXT |
| --- | --- | --- | --- | --- | --- | --- | --- | --- | --- | --- | --- | --- | --- | --- | --- | --- | --- | --- | --- | --- | --- | --- |
| Blackbird (n=6) | | |  |  |  |  |  |  |  |  |  |  |  |  |  |  |  |  |  |  |  |  |
| Resistant | 0,0,0 | 0,1,1 | 4,3,3 | 0,1,2 | 1,0,1 | 0,2,0 | 0,0,0 | 1,0,1 | 0,0,1 | 3,2,1 | 1,2,1 | 1,1,2 | 3,4,1 | 0,2,1 | 1,0,0 | 0,0,0 | 0,0,1 | 4,2,3 | 2,0,1 | 1,2,1 | 4,1,1 | 0,0,1 |
| Intermediate | 0,0,0 | 0,0,0 | 0,0,0 | 0,0,0 | 0,0,0 | 0,0,0 | 0,0,0 | 0,1,0 | 0,0,0 | 0,0,0 | 0,0,0 | 0,0,0 | 0,0,0 | 0,0,0 | 0,0,0 | 0,0,0 | 0,0,0 | 0,0,0 | 0,0,0 | 0,0,0 | 0,0,0 | 0,0,0 |
| Susceptible | 6,6,6 | 6,5,5 | 2,3,3 | 6,5,4 | 5,6,5 | 6,4,6 | 6,6,6 | 5,5,5 | 6,6,5 | 3,4,5 | 5,4,5 | 5,5,4 | 3,2,5 | 6,4,5 | 5,6,6 | 6,6,6 | 6,6,5 | 2,4,3 | 4,6,5 | 5,4,5 | 2,5,5 | 6,6,5 |
| Blackcap (n=25) | | | | | |  |  |  |  |  |  |  |  |  |  |  |  |  |  |  |  |  |
| Resistant | 0,1,0 | 3,4,5 | 13,13,12 | 0,3,1 | 4,1,3 | 0,1,2 | 0,2,3 | 5,3,3 | 0,0,1 | 8,7,3 | 3,7,4 | 8,3,9 | 11,6,9 | 2,4,1 | 2,0,3 | 1,0,0 | 0,1,1 | 13,10,6 | 4,7,5 | 8,4,1 | 11,8,9 | 2,1,2 |
| Intermediate | 0,0,1 | 0,0,2 | 2,0,0 | 0,0,0 | 0,0,0 | 0,0,0 | 0,0,0 | 0,2,0 | 0,0,0 | 0,0,0 | 1,0,2 | 0,0,2 | 0,0,1 | 0,0,3 | 0,1,1 | 0,1,0 | 0,0,0 | 1,0,2 | 0,1,0 | 0,0,1 | 1,0,0 | 0,0,0 |
| Susceptible | 25,24,24 | 22,21,18 | 10,12,13 | 25,22,24 | 21,24,22 | 25,24,23 | 25,23,22 | 20,20,22 | 25,25,24 | 17,18,22 | 21,18,19 | 17,22,14 | 14,19,15 | 23,21,21 | 23,24,21 | 24,24,25 | 25,24,24 | 11,15,17 | 21,17,20 | 17,21,23 | 13,17,16 | 23,24,23 |
| Blue tit (n=29) | | | | | |  |  |  |  |  |  |  |  |  |  |  |  |  |  |  |  |  |
| Resistant | 1,1,1 | 3,6,8 | 16,10,15 | 0,1,2 | 3,4,4 | 1,1,1 | 2,1,1 | 2,2,5 | 1,3,3 | 10,7,11 | 5,4,7 | 5,8,11 | 18,13,6 | 7,4,5 | 3,1,2 | 0,2,0 | 2,1,1 | 18,14,8 | 7,4,5 | 7,11,1 | 11,10,8 | 1,1,2 |
| Intermediate | 0,0,1 | 0,0,0 | 0,0,0 | 1,0,1 | 0,0,1 | 0,0,0 | 3,1,1 | 0,0,1 | 0,0,0 | 1,0,0 | 5,0,1 | 0,0,1 | 0,0,1 | 1,1,1 | 0,0,0 | 0,1,0 | 0,0,1 | 0,0,3 | 0,3,1 | 1,0,0 | 0,0,1 | 0,0,3 |
| Susceptible | 28,28,27 | 26,23,21 | 13,19,14 | 28,28,26 | 26,25,24 | 28,28,28 | 24,27,27 | 27,27,23 | 28,26,26 | 18,22,18 | 19,25,21 | 24,21,17 | 11,16,22 | 21,24,23 | 26,28,27 | 29,26,29 | 27,28,27 | 11,15,18 | 22,22,23 | 21,18,28 | 18,19,20 | 28,28,24 |
| Bullfinch (n=3) | | | | |  |  |  |  |  |  |  |  |  |  |  |  |  |  |  |  |  |  |
| Resistant | 0,0,0 | 0,0,0 | 0,1,1 | 0,0,0 | 0,0,0 | 0,0,0 | 0,0,0 | 0,0,0 | 0,0,0 | 2,0,1 | 0,0,0 | 1,0,0 | 1,0,0 | 0,0,0 | 0,1,0 | 0,0,0 | 0,0,0 | 1,1,0 | 1,0,0 | 0,0,1 | 0,0,0 | 0,0,1 |
| Intermediate | 0,0,0 | 0,0,0 | 0,0,0 | 0,1,0 | 0,0,0 | 0,0,0 | 0,0,0 | 0,0,0 | 0,0,0 | 0,0,0 | 0,0,0 | 0,0,0 | 0,0,0 | 0,0,0 | 0,0,0 | 0,0,0 | 0,0,0 | 0,0,0 | 0,0,0 | 0,0,0 | 0,0,0 | 0,0,0 |
| Susceptible | 3,3,3 | 3,3,3 | 3,2,2 | 3,2,3 | 3,3,3 | 3,3,3 | 3,3,3 | 3,3,3 | 3,3,3 | 1,3,2 | 3,3,3 | 2,3,3 | 2,3,3 | 3,3,3 | 3,2,3 | 3,3,3 | 3,3,3 | 2,2,3 | 2,3,3 | 3,3,2 | 3,3,3 | 3,3,2 |
| Chaffinch (n=30) | | | | | |  |  |  |  |  |  |  |  |  |  |  |  |  |  |  |  |  |
| Resistant | 0,2,0 | 6,4,6 | 14,13,14 | 2,0,3 | 2,5,7 | 1,0,3 | 0,0,3 | 2,6,4 | 1,2,1 | 13,8,11 | 7,7,5 | 7,8,7 | 17,11,8 | 8,5,3 | 4,5,3 | 0,0,0 | 3,3,2 | 17,15,13 | 7,4,10 | 9,8,2 | 15,9,10 | 0,3,6 |
| Intermediate | 0,0,0 | 0,0,0 | 3,0,0 | 0,0,0 | 0,0,0 | 0,2,0 | 3,0,0 | 0,0,1 | 0,0,0 | 1,1,0 | 1,0,3 | 0,1,2 | 1,0,1 | 2,1,1 | 0,2,0 | 0,0,0 | 0,0,0 | 2,0,0 | 0,0,0 | 0,0,0 | 2,0,1 | 2,0,1 |
| Susceptible | 30,28,30 | 24,26,24 | 13,17,16 | 28,30,27 | 28,25,23 | 29,28,27 | 27,30,27 | 28,24,25 | 29,28,29 | 16,21,19 | 22,23,22 | 23,21,21 | 12,19,21 | 20,24,26 | 26,23,27 | 30,30,30 | 27,27,28 | 11,15,17 | 23,26,20 | 21,22,28 | 13,21,19 | 28,27,23 |
| Chiffchaff (n=3) | | | |  |  |  |  |  |  |  |  |  |  |  |  |  |  |  |  |  |  |  |
| Resistant | 0,0,0 | 1,2,1 | 1,2,2 | 0,0,0 | 0,0,0 | 0,0,0 | 0,0,0 | 1,0,0 | 0,0,0 | 1,1,1 | 2,0,1 | 2,1,1 | 2,1,1 | 1,1,2 | 0,0,1 | 0,0,0 | 0,0,0 | 2,2,2 | 2,2,1 | 0,1,0 | 1,1,2 | 0,0,0 |
| Susceptible | 3,3,3 | 2,1,2 | 2,1,1 | 3,3,3 | 3,3,3 | 3,3,3 | 3,3,3 | 2,3,3 | 3,3,3 | 2,2,2 | 1,3,2 | 1,2,2 | 1,2,2 | 2,2,1 | 3,3,2 | 3,3,3 | 3,3,3 | 1,1,1 | 1,1,2 | 3,2,3 | 2,2,1 | 3,3,3 |
| Dunnock (n=14) | | | | | |  |  |  |  |  |  |  |  |  |  |  |  |  |  |  |  |  |
| Resistant | 0,1,1 | 2,1,3 | 8,6,7 | 0,0,0 | 3,3,2 | 3,0,0 | 0,2,0 | 5,2,3 | 0,0,0 | 2,6,2 | 3,5,5 | 0,5,3 | 8,6,5 | 3,0,3 | 2,1,2 | 0,0,0 | 0,0,1 | 6,5,7 | 3,3,2 | 4,3,5 | 6,5,4 | 3,1,2 |
| Intermediate | 0,0,0 | 0,0,0 | 1,0,0 | 0,0,0 | 0,0,0 | 0,1,0 | 1,1,0 | 0,1,1 | 0,0,0 | 0,0,0 | 3,0,0 | 0,1,2 | 0,0,0 | 2,0,0 | 0,0,0 | 0,0,0 | 0,0,0 | 0,0,0 | 0,0,0 | 0,0,0 | 0,0,0 | 0,0,0 |
| Susceptible | 14,13,13 | 12,13,11 | 5,8,7 | 14,14,14 | 11,11,12 | 11,13,14 | 13,11,14 | 9,11,10 | 14,14,14 | 12,8,12 | 8,9,9 | 14,8,9 | 6,8,9 | 9,14,11 | 12,13,12 | 14,14,14 | 14,14,13 | 8,9,7 | 11,11,12 | 10,11,9 | 8,9,10 | 11,13,12 |
| Garden warbler (n=1) | | | | |  |  |  |  |  |  |  |  |  |  |  |  |  |  |  |  |  |  |
| Resistant | 0,0,0 | 0,0,0 | 1,0,0 | 0,0,0 | 1,0,1 | 0,0,0 | 0,0,0 | 0,0,0 | 0,0,0 | 0,0,1 | 0,1,0 | 0,0,0 | 0,0,0 | 0,0,0 | 0,0,0 | 0,0,0 | 0,0,0 | 1,0,1 | 0,0,0 | 0,0,0 | 1,1,0 | 0,0,1 |
| Susceptible | 1,1,1 | 1,1,1 | 0,1,1 | 1,1,1 | 0,1,0 | 1,1,1 | 1,1,1 | 1,1,1 | 1,1,1 | 1,1,0 | 1,0,1 | 1,1,1 | 1,1,1 | 1,1,1 | 1,1,1 | 1,1,1 | 1,1,1 | 0,1,0 | 1,1,1 | 1,1,1 | 0,0,1 | 1,1,0 |
| Goldfinch (n=4) | | | | | |  |  |  |  |  |  |  |  |  |  |  |  |  |  |  |  |  |
| Resistant | 0,1,0 | 0,1,0 | 1,3,2 | 0,0,1 | 1,0,1 | 0,0,0 | 0,0,1 | 0,0,1 | 0,0,0 | 2,2,1 | 1,1,0 | 1,2,0 | 2,1,1 | 0,0,2 | 1,1,1 | 0,0,0 | 0,0,0 | 1,1,1 | 2,0,2 | 0,2,1 | 1,1,1 | 1,0,0 |
| Intermediate | 0,0,0 | 0,0,0 | 0,0,0 | 0,0,0 | 0,0,0 | 0,1,0 | 0,0,0 | 0,0,0 | 0,0,0 | 0,0,0 | 0,0,1 | 0,0,0 | 0,0,0 | 0,1,0 | 0,0,0 | 0,0,0 | 0,0,0 | 0,0,0 | 0,0,0 | 0,0,0 | 0,0,0 | 0,0,0 |
| Susceptible | 4,3,4 | 4,3,4 | 3,1,2 | 4,4,3 | 3,4,3 | 4,3,4 | 4,4,3 | 4,4,3 | 4,4,4 | 2,2,3 | 3,3,3 | 3,2,4 | 2,3,3 | 4,3,2 | 3,3,3 | 4,4,4 | 4,4,4 | 3,3,3 | 2,4,2 | 4,2,3 | 3,3,3 | 3,4,4 |
| Great tit (n=13) | | | | |  |  |  |  |  |  |  |  |  |  |  |  |  |  |  |  |  |  |
| Resistant | 0,0,1 | 2,1,2 | 5,5,4 | 2,2,1 | 3,3,4 | 0,0,1 | 0,0,1 | 3,3,3 | 1,1,2 | 4,6,2 | 4,4,5 | 4,3,4 | 4,3,1 | 3,2,1 | 1,4,4 | 1,0,0 | 2,1,1 | 6,4,2 | 3,5,2 | 3,6,6 | 4,4,7 | 2,0,0 |
| Intermediate | 0,0,1 | 0,0,0 | 2,0,0 | 0,0,0 | 0,0,2 | 0,1,0 | 1,0,0 | 0,0,1 | 0,0,0 | 0,1,0 | 0,0,0 | 0,0,0 | 3,0,1 | 0,1,0 | 0,1,0 | 0,1,0 | 0,0,0 | 0,0,4 | 0,0,0 | 0,0,0 | 0,0,0 | 1,0,0 |
| Susceptible | 13,13,11 | 11,12,11 | 6,8,9 | 11,11,12 | 10,10,7 | 13,12,12 | 12,13,12 | 10,10,9 | 12,12,11 | 9,6,11 | 9,9,8 | 9,10,9 | 6,10,11 | 10,10,12 | 12,8,9 | 12,12,13 | 12,12,12 | 7,9,7 | 10,8,11 | 10,7,7 | 9,9,6 | 10,13,13 |
| Greenfinch (n=4) | | | | | |  |  |  |  |  |  |  |  |  |  |  |  |  |  |  |  |  |
| Resistant | 0,0,1 | 1,0,0 | 1,1,1 | 0,0,0 | 0,0,0 | 0,0,0 | 0,0,0 | 0,0,0 | 0,0,0 | 1,1,1 | 0,0,0 | 1,0,0 | 1,0,0 | 0,0,0 | 0,0,0 | 0,0,0 | 0,0,0 | 0,1,0 | 0,0,0 | 0,0,1 | 1,1,1 | 0,1,0 |
| Intermediate | 0,0,0 | 0,0,0 | 1,0,0 | 0,1,0 | 0,0,0 | 0,0,0 | 0,0,0 | 0,0,1 | 0,0,0 | 1,0,0 | 2,0,0 | 0,0,0 | 0,0,1 | 0,0,0 | 0,0,0 | 0,0,0 | 0,0,0 | 0,0,0 | 0,1,0 | 0,0,0 | 0,0,0 | 1,0,0 |
| Susceptible | 4,4,3 | 3,4,4 | 2,3,3 | 4,3,4 | 4,4,4 | 4,4,4 | 4,4,4 | 4,4,3 | 4,4,4 | 2,3,3 | 2,4,4 | 3,4,4 | 3,4,3 | 4,4,4 | 4,4,4 | 4,4,4 | 4,4,4 | 4,3,4 | 4,3,4 | 4,4,3 | 3,3,3 | 3,3,4 |
| House sparrow (n=40) | | | | | | |  |  |  |  |  |  |  |  |  |  |  |  |  |  |  |  |
| Resistant | 1,1,3 | 5,5,4 | 14,18,15 | 1,2,2 | 3,6,3 | 1,3,5 | 1,1,1 | 5,6,5 | 0,2,0 | 10,7,15 | 6,10,6 | 10,5,7 | 13,7,5 | 5,4,2 | 4,0,6 | 2,1,2 | 2,2,3 | 12,18,15 | 10,4,10 | 7,10,10 | 15,13,11 | 5,8,3 |
| Intermediate | 0,0,1 | 0,1,0 | 0,0,0 | 1,1,0 | 0,0,0 | 0,2,0 | 0,0,0 | 0,1,0 | 0,0,0 | 2,2,0 | 3,0,1 | 0,0,1 | 0,1,0 | 1,1,2 | 0,0,0 | 0,0,0 | 0,0,0 | 0,0,0 | 0,2,0 | 0,0,2 | 0,0,0 | 0,1,1 |
| Susceptible | 39,39,36 | 35,34,36 | 26,22,25 | 38,37,38 | 37,34,37 | 39,35,35 | 39,39,39 | 35,33,35 | 40,38,40 | 28,31,25 | 31,30,33 | 30,35,32 | 27,32,35 | 34,35,36 | 36,40,34 | 38,39,38 | 38,38,37 | 28,22,25 | 30,34,30 | 33,30,28 | 25,27,29 | 35,31,36 |
| Lesser whitethroat (n=1) | | | | | | |  |  |  |  |  |  |  |  |  |  |  |  |  |  |  |  |
| Resistant | 0,0,0 | 0,0,0 | 0,0,0 | 0,0,0 | 0,0,0 | 0,0,0 | 0,0,0 | 0,0,0 | 0,0,0 | 0,0,0 | 0,0,0 | 0,0,0 | 0,0,0 | 0,0,0 | 0,0,0 | 0,0,0 | 0,0,0 | 0,0,0 | 0,0,0 | 0,0,0 | 0,0,0 | 0,0,0 |
| Susceptible | 1,1,1 | 1,1,1 | 1,1,1 | 1,1,1 | 1,1,1 | 1,1,1 | 1,1,1 | 1,1,1 | 1,1,1 | 1,1,1 | 1,1,1 | 1,1,1 | 1,1,1 | 1,1,1 | 1,1,1 | 1,1,1 | 1,1,1 | 1,1,1 | 1,1,1 | 1,1,1 | 1,1,1 | 1,1,1 |
| Long-tailed tit (n=3) | | | | | | |  |  |  |  |  |  |  |  |  |  |  |  |  |  |  |  |
| Resistant | 0,0,0 | 0,1,1 | 3,1,1 | 0,0,0 | 0,1,0 | 0,0,0 | 0,0,0 | 0,1,1 | 0,0,0 | 1,1,1 | 0,1,0 | 0,1,1 | 2,2,1 | 0,0,0 | 0,0,0 | 0,0,0 | 0,1,0 | 3,1,1 | 0,1,1 | 2,1,0 | 2,0,2 | 0,0,0 |
| Intermediate | 0,0,0 | 0,0,0 | 0,0,0 | 0,0,0 | 0,0,0 | 0,0,0 | 0,0,0 | 0,0,0 | 1,0,0 | 0,0,0 | 0,0,2 | 0,0,0 | 0,0,0 | 0,0,1 | 0,1,0 | 0,0,0 | 0,0,0 | 0,0,0 | 0,0,0 | 0,0,2 | 0,0,0 | 0,0,1 |
| Susceptible | 3,3,3 | 3,2,2 | 0,2,2 | 3,3,3 | 3,2,3 | 3,3,3 | 3,3,3 | 3,2,2 | 2,3,3 | 2,2,2 | 3,2,1 | 3,2,2 | 1,1,2 | 3,3,2 | 3,2,3 | 3,3,3 | 3,2,3 | 0,2,2 | 3,2,2 | 1,2,1 | 1,3,1 | 3,3,2 |
| Reed bunting (n=5) | | | | | |  |  |  |  |  |  |  |  |  |  |  |  |  |  |  |  |  |
| Resistant | 0,0,0 | 2,1,1 | 3,4,2 | 1,1,0 | 1,1,1 | 0,0,1 | 0,0,1 | 2,2,1 | 0,0,0 | 1,1,1 | 1,1,0 | 0,2,1 | 4,2,1 | 0,1,3 | 0,0,0 | 0,0,0 | 0,0,0 | 3,2,2 | 2,1,0 | 3,1,0 | 2,1,1 | 0,0,1 |
| Intermediate | 0,0,1 | 0,0,0 | 0,0,0 | 0,0,0 | 0,0,0 | 0,0,1 | 0,0,0 | 0,0,0 | 0,0,0 | 0,0,0 | 1,0,1 | 0,0,0 | 0,0,0 | 1,0,0 | 0,0,0 | 0,0,0 | 0,0,0 | 0,0,1 | 0,0,0 | 0,0,0 | 0,0,0 | 0,0,0 |
| Susceptible | 5,5,4 | 3,4,4 | 2,1,3 | 4,4,5 | 4,4,4 | 5,5,3 | 5,5,4 | 3,3,4 | 5,5,5 | 4,4,4 | 3,4,4 | 5,3,4 | 1,3,4 | 4,4,2 | 5,5,5 | 5,5,5 | 5,5,5 | 2,3,2 | 3,4,5 | 2,4,5 | 3,4,4 | 5,5,4 |
| Reed warbler (n=10) | | | | | |  |  |  |  |  |  |  |  |  |  |  |  |  |  |  |  |  |
| Resistant | 0,0,0 | 2,2,2 | 3,5,2 | 0,0,0 | 3,1,1 | 0,0,1 | 0,0,1 | 1,2,0 | 0,0,1 | 3,0,2 | 5,2,1 | 0,2,3 | 4,2,3 | 0,1,1 | 1,2,1 | 0,0,1 | 0,0,0 | 4,4,3 | 2,1,2 | 4,3,0 | 3,2,3 | 0,0,2 |
| Intermediate | 0,0,0 | 0,0,0 | 1,0,0 | 0,0,0 | 0,0,0 | 0,0,0 | 0,0,0 | 0,0,0 | 0,0,0 | 0,1,0 | 0,0,0 | 0,0,1 | 0,0,0 | 0,0,0 | 0,0,0 | 0,0,0 | 0,0,0 | 0,0,1 | 0,0,0 | 0,0,0 | 0,0,0 | 0,0,0 |
| Susceptible | 10,10,10 | 8,8,8 | 6,5,8 | 10,10,10 | 7,9,9 | 10,10,9 | 10,10,9 | 9,8,10 | 10,10,9 | 7,9,8 | 5,8,9 | 10,8,6 | 6,8,7 | 10,9,9 | 9,8,9 | 10,10,9 | 10,10,10 | 6,6,6 | 8,9,8 | 6,7,10 | 7,8,7 | 10,10,8 |
| Robin (n=24) |  |  |  |  |  |  |  |  |  |  |  |  |  |  |  |  |  |  |  |  |  |  |
| Resistant | 0,1,0 | 3,6,4 | 14,10,14 | 3,2,1 | 4,6,8 | 1,1,4 | 1,0,1 | 3,5,2 | 0,0,0 | 9,3,5 | 8,9,7 | 8,6,6 | 13,7,6 | 3,5,1 | 2,2,1 | 1,0,1 | 1,1,3 | 13,14,13 | 8,7,7 | 9,6,6 | 14,9,9 | 2,2,4 |
| Intermediate | 0,0,0 | 0,0,0 | 1,0,0 | 0,0,0 | 0,0,0 | 0,2,1 | 1,0,0 | 0,0,0 | 1,0,0 | 1,3,0 | 4,0,0 | 0,0,2 | 1,0,2 | 4,0,2 | 0,3,2 | 0,0,0 | 0,1,0 | 0,0,2 | 0,1,0 | 1,0,5 | 0,0,0 | 3,0,0 |
| Susceptible | 24,23,24 | 21,18,20 | 9,14,10 | 21,22,23 | 20,18,16 | 23,21,19 | 22,24,23 | 21,19,22 | 23,24,24 | 14,18,19 | 12,15,17 | 16,18,16 | 10,17,16 | 17,19,21 | 22,19,21 | 23,24,23 | 23,22,21 | 11,10,9 | 16,16,17 | 14,18,13 | 10,15,15 | 19,22,20 |
| Sedge warbler (n=3) | | | | | |  |  |  |  |  |  |  |  |  |  |  |  |  |  |  |  |  |
| Resistant | 0,0,0 | 0,0,1 | 3,1,2 | 0,0,0 | 0,1,0 | 0,0,0 | 0,0,0 | 1,1,2 | 1,0,0 | 3,0,0 | 0,1,0 | 0,1,1 | 3,3,3 | 0,1,3 | 0,0,0 | 0,0,0 | 0,1,0 | 3,3,3 | 1,0,1 | 0,2,0 | 3,2,1 | 1,0,0 |
| Intermediate | 0,0,0 | 0,0,0 | 0,0,0 | 0,0,0 | 0,0,0 | 0,0,0 | 0,0,0 | 0,0,0 | 0,0,0 | 0,0,0 | 1,0,1 | 0,0,0 | 0,0,0 | 0,0,0 | 0,0,0 | 0,0,0 | 0,0,0 | 0,0,0 | 0,1,0 | 1,0,0 | 0,0,0 | 0,0,0 |
| Susceptible | 3,3,3 | 3,3,2 | 0,2,1 | 3,3,3 | 3,2,3 | 3,3,3 | 3,3,3 | 2,2,1 | 2,3,3 | 0,3,3 | 2,2,2 | 3,2,2 | 0,0,0 | 3,2,0 | 3,3,3 | 3,3,3 | 3,2,3 | 0,0,0 | 2,2,2 | 2,1,3 | 0,1,2 | 2,3,3 |
| Song thrush (n=1) | | | | | |  |  |  |  |  |  |  |  |  |  |  |  |  |  |  |  |  |
| Resistant | 0,0,0 | 0,0,0 | 0,0,1 | 0,0,0 | 0,0,0 | 0,0,0 | 0,0,0 | 0,0,0 | 0,0,0 | 0,0,0 | 0,0,0 | 0,0,0 | 0,1,0 | 0,0,0 | 0,0,0 | 0,0,0 | 0,0,0 | 0,0,0 | 0,0,0 | 0,0,0 | 0,0,0 | 0,0,0 |
| Susceptible | 1,1,1 | 1,1,1 | 1,1,0 | 1,1,1 | 1,1,1 | 1,1,1 | 1,1,1 | 1,1,1 | 1,1,1 | 1,1,1 | 1,1,1 | 1,1,1 | 1,0,1 | 1,1,1 | 1,1,1 | 1,1,1 | 1,1,1 | 1,1,1 | 1,1,1 | 1,1,1 | 1,1,1 | 1,1,1 |
| Whitethroat (n=15) | | | | | |  |  |  |  |  |  |  |  |  |  |  |  |  |  |  |  |  |
| Resistant | 0,0,0 | 2,2,0 | 5,6,6, | 1,1,1 | 1,1,1 | 2,1,0 | 0,0,1 | 2,1,2 | 1,0,0 | 4,5,0 | 1,3,3 | 3,2,3 | 5,1,1 | 1,3,1 | 0,0,1 | 0,0,1 | 0,0,1 | 4,4,4 | 4,0,2 | 3,3,3 | 4,5,5 | 1,2,3 |
| Intermediate | 0,0,0 | 0,0,0 | 0,0,0 | 0,0,0 | 0,0,0 | 0,0,0 | 0,0,0 | 0,0,0 | 0,0,0 | 0,0,0 | 0,0,0 | 0,0,0 | 0,0,0 | 0,0,0 | 0,1,0 | 0,0,0 | 0,0,0 | 0,0,0 | 0,0,0 | 1,0,0 | 0,0,0 | 1,0,0 |
| Susceptible | 15,15,15 | 13,13,15 | 10,9,9 | 14,14,14 | 14,14,14 | 13,14,15 | 15,15,14 | 13,14,13 | 14,15,15 | 11,10,15 | 14,12,12 | 12,13,12 | 10,14,14 | 14,12,14 | 15,14,14 | 15,15,14 | 15,15,14 | 11,11,11 | 11,15,13 | 11,12,12 | 11,10,10 | 13,13,12 |
| Willow warbler (n=5) | | | | | |  |  |  |  |  |  |  |  |  |  |  |  |  |  |  |  |  |
| Resistant | 0,0,0 | 0,1,0 | 2,2,3 | 0,0,0 | 0,1,.1 | 1,0,0 | 0,0,1 | 0,1,1 | 1,0,0 | 2,1,1 | 1,2,1 | 1,1,1 | 2,1,1 | 1,1,0 | 0,0,1 | 0,0,0 | 1,1,0 | 2,2,0 | 2,0,2 | 1,1,1 | 2,2,1 | 0,0,1 |
| Intermediate | 0,0,0 | 0,0,0 | 0,0,0 | 0,0,0 | 0,0,0 | 0,0,0 | 0,0,0 | 0,0,0 | 0,0,0 | 0,0,0 | 0,0,0 | 0,0,0 | 0,0,0 | 0,0,0 | 0,0,0 | 0,0,0 | 0,0,0 | 0,0,0 | 0,0,0 | 0,0,0 | 0,0,0 | 1,0,0 |
| Susceptible | 5,5,5 | 5,4,5 | 3,3,2 | 5,5,5 | 5,4,4 | 4,5,5 | 5,5,4 | 5,4,4 | 4,5,5 | 3,4,4 | 4,3,4 | 4,4,4 | 3,4,4 | 4,4,5 | 5,5,4 | 5,5,5 | 4,4,5 | 3,3,5 | 3,5,3 | 4,4,4 | 3,3,4 | 4,5,4 |
| Wren (n=16) |  |  |  |  |  |  |  |  |  |  |  |  |  |  |  |  |  |  |  |  |  |  |
| Resistant | 0,0,0 | 2,3,2 | 3,5,6 | 2,1,2 | 2,1,4 | 2,0,0 | 0,1,1 | 4,1,2 | 0,0,0 | 8,3,3 | 4,4,3 | 4,4,5 | 7,1,5 | 2,2,2 | 1,2,0 | 1,0,0 | 0,0,0 | 4,6,4 | 4,5,1 | 4,2,3 | 4,6,5 | 1,0,1 |
| Intermediate | 0,0,1 | 0,0,0 | 2,0,0 | 0,0,0 | 0,0,0 | 0,2,0 | 0,1,0 | 0,1,0 | 2,0,0 | 0,1,0 | 2,0,0 | 0,0,0 | 0,0,0 | 0,0,0 | 0,1,0 | 0,1,0 | 0,0,0 | 1,0,1 | 0,1,0 | 2,0,0 | 0,0,0 | 0,0,0 |
| Susceptible | 16,16,15 | 14,13,14 | 11,11,10 | 14,15,14 | 14,15,12 | 14,14,16 | 16,14,15 | 12,14,14 | 14,16,16 | 8,12,13 | 10,12,13 | 12,12,11 | 9,15,11 | 14,14,14 | 15,13,16 | 15,15,16 | 16,16,16 | 11,10,11 | 12,10,15 | 10,14,13 | 12,10,11 | 15,16,15 |
| Yellowhammer (n=4) | | | | |  |  |  |  |  |  |  |  |  |  |  |  |  |  |  |  |  |  |
| Resistant | 0,0,0 | 2,0,3 | 3,2,1 | 0,0,0 | 0,0,0 | 0,0,0 | 0,0,0 | 0,0,1 | 0,0,0 | 1,0,3 | 1,0,0 | 1,2,1 | 2,2,2 | 1,2,0 | 0,1,0 | 0,0,0 | 0,0,0 | 2,2,1 | 0,2,0 | 1,2,1 | 3,1,0 | 0,0,1 |
| Intermediate | 0,0,0 | 0,0,0 | 0,0,1 | 0,0,0 | 0,0,0 | 0,0,0 | 0,0,0 | 0,0,1 | 0,0,0 | 0,1,0 | 1,0,1 | 0,0,1 | 0,0,0 | 0,0,0 | 0,0,0 | 0,0,0 | 0,0,0 | 0,0,0 | 0,0,1 | 0,0,0 | 0,0,0 | 0,0,0 |
| Susceptible | 4,4,4 | 2,4,1 | 1,2,2 | 4,4,4 | 4,4,4 | 4,4,4 | 4,4,4 | 4,4,2 | 4,4,4 | 3,3,1 | 2,4,3 | 3,2,2 | 2,2,2 | 3,2,4 | 4,3,4 | 4,4,4 | 4,4,4 | 2,2,3 | 4,2,3 | 3,2,3 | 1,3,4 | 4,4,3 |
| Total (n=259) Mean ± SE | | | | |  |  |  |  |  |  |  |  |  |  |  |  |  |  |  |  |  |  |
| Resistant | 5.7±1.9 | 40.3 ±2.3 | 114±1.7 | 14±1.2 | 36.3±3.0 | 13±2.6 | 9±3.6 | 36.7±0.3 | 7.7±0.9 | 71.7±8.3 | 55.3±4.5 | 60.0±3.0 | 85.3±18.8 | 35.3±2.2 | 22.7±1.8 | 4.7±0.9 | 12.3±0.9 | 106±9.0 | 54.7±5.2 | 59.0±8.0 | 90.0±8.5 | 23.0±4.0 |
| Intermediate | 2.0±2.0 | 1±0.6 | 4.7±4.2 | 4.7±4.2 | 1.0±1.0 | 4.3±3.4 | 4.3±2.4 | 4.0±2.0 | 1.3±1.3 | 5.3±2.9 | 12.3±6.9 | 4.7±3.7 | 4.3±1.8 | 8.7±1.9 | 4.3±3.0 | 1.3±1.3 | 0.7±0.3 | 6.0±4.2 | 4.0±3.1 | 5.3±2.9 | 1.7±0.9 | 5.3±2.3 |
| Susceptible | 251±3.2 | 218±2.9 | 140±5.8 | 243±1 | 222±3.9 | 242±2.7 | 246±2.0 | 218±1.9 | 250±0.6 | 182±8.6 | 191±4.7 | 194±6.7 | 169±18.8 | 215±2.1 | 232±2.5 | 253±0.6 | 246±1.2 | 147±5.8 | 200±2.7 | 195±5.8 | 231±4.9 | 230±4.0 |

AK: Amikacin (30 mg/ml); AMC: Amoxicillin-clavulanic acid (20mg); AMP: Ampicillin (10 mg/ml); AZ: Aztreonam (30 mg/ml); CTX: Cefotaxime (30 mg/ml); FOX: Cefoxitin (30 mg/ml); CAZ: Ceftazidime (30 mg/ml); CFT: Ceftiofur (30 mg/ml); CRO: Ceftriaxone (30 mg/ml); C: Chloramphenicol (30 mg/ml); CIP: Ciprofloxacin (5 mg/ml); ENR: Enrofloxacin (5 mg/ml); E: Erythromycin (15 mg/ml); CN: Gentamicin (10 mg/ml); IMP: Imipoenem (10 mg/ml); K: Kanamycin (30 mg/ml); M: Meropenem (10 mg/ml); NA: Nalidixic acid (30 mg/ml); S: Streptomycin (10 mg/ml); TE: Tetracyclin (30 mg/ml); Ti: Ticarcillin (75 mg/ml); SXT: Trimethoprim-sulfamethoxazole (20 mg/ml)
